# Global molecular epidemiology and genetic diversity of *Fusarium*, a significant emerging group of human opportunists from 1958 to 2015

**DOI:** 10.1038/emi.2016.126

**Published:** 2016-12-07

**Authors:** Abdullah MS Al-Hatmi, Ferry Hagen, Steph BJ Menken, Jacques F Meis, G Sybren de Hoog

**Affiliations:** 1 CBS-KNAW Fungal Biodiversity Centre, Department of Medical Mycology, Utrecht 3508 AD, The Netherlands; 2 Institutes of Biodiversity and Ecosystem Dynamics, Department of Biology, University of Amsterdam, Amsterdam 1098 XH, The Netherlands; 3 Directorate General of Health Services, Ibri Hospital, Department of laboratories, Ministry of Health, Muscat PC 113, Oman; 4 Department of Medical Microbiology and Infectious Diseases, Canisius-Wilhelmina Hospital, Nijmegen 6532 SZ, The Netherlands; 5 Department of Medical Microbiology, Radboudumc, Nijmegen 6525 HP, The Netherlands; 6 Department of Basic Pathology, Federal University of Parana State, Curitiba 81540-970, Parana, Brazil; 7 Department of Biology, Faculty of Science, King Abdulaziz University, Jeddah 21589, Saudi Arabia

**Keywords:** AFLP, fusariosis, *Fusarium*, genotyping, molecular epidemiology

## Abstract

*Fusarium* is a rapidly emerging, multidrug-resistant genus of fungal opportunists that was first identified in 1958 and is presently recognized in numerous cases of fusariosis each year. The authors examined trends in global *Fusarium* distribution, clinical presentation and prevalence since 1958 with the assumption that their distributions in each region had remained unaltered. The phylogeny and epidemiology of 127 geographically diverse isolates, representing 26 *Fusarium* species, were evaluated using partial sequences of the *RPB2* and *TEF1* genes, and compared with AFLP fingerprinting data. The molecular data of the *Fusarium* species were compared with archived data, which enabled the interpretation of hundreds of cases published in the literature. Our findings indicate that fusariosis is globally distributed with a focus in (sub)tropical areas. Considerable species diversity has been observed; genotypic features did not reveal any clustering with either the clinical data or environmental origins. This study suggests that infections with *Fusarium* species might be truly opportunistic. The three most common species are *F. falciforme* and *F. keratoplasticum* (members of *F. solani* species complex), followed by *F. oxysporum* (*F. oxysporum* species complex).

## INTRODUCTION

*Fusarium* infections are a major challenge with respect to the diagnosis and treatment, especially in neutropenic patients. Disseminated infections may be fatal and are a considerable source of increased healthcare costs. A major area of concern is the intrinsic resistance to a broad range of antifungals,^1^ which is a characteristic of *Fusarium*. During the past decade, the *F. solani* complex has received special interest because of the increasing numbers of infections worldwide.^2^ More than 300 cases of *Fusarium* keratitis were associated with contaminated contact lens cleaning solution, causing outbreaks between 2005 and 2007, where members of the *F*. *solani* species complex played a major role.^3^ Furthermore, reservoirs of infectious *Fusarium* species in hospital environments, especially plumbing and water systems, have been reported.^4^

Although human fusariosis was only recognized since the late 1950s and endemic areas are mostly located in tropical and subtropical countries,^5^ their global significance has only recently come into focus within the past three decades. Etiological agents differ in antifungal susceptibility,^6^ virulence profiles, geographic distribution, ecological niches, life cycle, host and mycotoxin production.^7^ Although agents of fusariosis are mostly environmental,^8^ the potential of nosocomial transmission has recently been raised,^9^ especially with reference to the high mortality rate of ~90% in patients with prolonged and severe neutropenia.^10^

The burden of disease has not been established, but numerous clinical case series and case reports provide an estimate of the magnitude of the problem. Most published studies have focused on prevalence in single healthcare centers.^10, 11, 12, 13, 14, 15, 16^ Nucci *et al.*^17^ reported 233 cases from different hospitals on a global scale. Mohammed *et al.*^18^ reported 26 cases from the United States and reviewed 97 cases from the literature, and Horn *et al.*^12^ described 65 cases from the North American Path Alliance Registry. A major problem in comparative studies is the subdivision of the classical species into a series of molecular siblings, which renders the older literature without sequence data uninterpretable. Despite the current clinical importance of the organism, the phylogenetic relationships among species, varieties and geographical groups in *Fusarium* are currently elusive. Hence, the re-interpretation of these data in the light of modern molecular phylogeny is compulsory.

Molecular phylogenetic studies have led to the description of many *Fusarium* species with clinical relevance. These include members of the *F. solani* species complex, namely, *F. falciforme*, *F. keratoplasticum*, *F. lichenicola*, *F. petroliphilum*, *F. pseudensiforme* and *F. solani* (FSSC5), which is also known as *Fusisporium solani* and *Fusarium* haplotype ‘6'. The *F. oxysporum* species complex (FOSC) contains three lineages, which are involved in fusariosis and still have not been formally introduced as taxonomic species. The *F. fujikuroi* species complex includes *F. acutatum*, *F. ananatum*, *F. anthophilum*, *F. andiyazi*, *F. fujikuroi s.s.*, *F. globosum*, *F. guttiforme*, *F. musae*, *F. napiforme*, *F. nygamai*, *F. verticillioides*, *F. proliferatum*, *F. ramigenum*, *F. sacchari*, *F. subglutinans*, *F. temperatum* and *F. thapsinum*. Although rare, species of other *Fusarium* lineages are emerging as potential opportunistic pathogens, for example, in the *F. incarnatum-equiseti* species complex (FIESC; *F. incarnatum* and *F. equiseti*), the *F. dimerum* species complex (*F. dimerum*, *F. delphiniodes* and *F. penzigii*), the *F. chlamydosporum* species complex, the *F. sambucinum* species complex (*F. armeniacum*, *F. brachygibbosum*, *F. langsethiae* and *F. sporotrichioides*) and the *F. tricictum* species complex (*F. acuminatum* and *F. flocciferum*).^1^

Over the past decade, the number of cases of fusariosis has increased worldwide, but there are only a few reports describing the molecular epidemiology; therefore, the aim of the present study is to introduce a hypothetical system that permits the interpretation and use of at least a part of the literature where sequence data are lacking. Pre-molecular publications, which include interpretable case reports and geographical information, were collected. Subsequently, available *Fusarium* strains that were collected worldwide and deposited during the past century in the CBS-KNAW, Fungal Biodiversity Centre, culture collection Utrecht, The Netherlands, were sequenced and re-identified with current diagnostic technology, which enables the phylogenetic analysis of the human–pathogenic *Fusarium* species. These data were then compared with published materials and their distribution with the assumption that their distributions in each region had remained unaltered.

## MATERIALS AND METHODS

### Fungal strains

A total of 127 strains collected from clinical samples (*n*=74; 58.3% collected between 1978 and 2015) and strains collected from the environment (*n*=53; 41.7% collected between 1929 and 2015) were analyzed. All of the strains were maintained under the name ‘*Fusarium*' in the reference collection of CBS-KNAW, Utrecht, the Netherlands. The data regarding geographic origins and sources of isolation are listed in Table 1. All of the available type strains were included. Stock cultures were maintained on slants of 2% malt extract agar at 24 °C. The strains were assigned to a clinical subgroup and an environment subgroup.

### DNA extraction

DNA was extracted following the Quick Cetyl trimethylammonium bromide (CTAB) protocol. A total of 1–10 mm^3^ fungal material was transferred to 2- mL screw-capped tubes prefilled with 490 μL 2 × CTAB buffer and 6–10 acid-washed glass beads. A total of 10 μL of proteinase K was added and mixed thoroughly on a MoBio vortex (MO BIO Laboratories, Inc., Carlsbad, CA, USA) for 10 min. Then, 500 μL of chloroform:isoamylalcohol (24:1) was added and shaken for 2 min after incubation for 60 min at 60 °C. The tubes were centrifuged for 10 min at 14 000 rpm, and the supernatant was collected in a new Eppendorf tube. To ~400 μL of the DNA sample, 2/3 vol (~270 μL) of ice-cold isopropanol was added and centrifuged again at 14 000 rpm for 10 min, and the upper layer was dissolved in 1 mL ice-cold 70% ethanol. The tubes were centrifuged again at 14 000 rpm for 2 min, air-dried and resuspended in 50 μL TE buffer. The quality of the genomic DNA was verified by running 2–3 μL on a 0.8% agarose gel. Then, the DNA was quantified with a NanoDrop 2000 spectrophotometer (Thermo Fisher, Wilmington, DE, USA), and the samples were stored at −20 °C until ready for analysis.

### DNA amplification and sequencing

The following two gene regions were amplified directly from the genomic DNA: the second largest subunit of RNA polymerase (*RPB2*; Reeb *et al.*^19^) and the translation elongation factor-1α (*TEF1α* O'Donnell *et al.*^20^) were amplified and sequenced following the methods published by Saleh *et al.*^16^ The PCR reactions were performed in a volume of 12.5 μL containing 1.25 μL of 10 × PCR buffer, 7.5 μL of water, 0.5 μL of dNTP mix (2.5 mM), 0.25 μL of each primer (10 pmol), 0.05 μL of Taq polymerase (5 U/μL), 0.7 μL of dimethylsulphoxide and 1 μL of template DNA (100 ng/μL). The amplification was performed with the ABI Prism 2720 thermal cycler (Applied Biosystems, Foster City, CA, USA). The cycling conditions included 1 cycle of 5 min at 94 °C, 10 cycles of 45 s at 94 °C, 45 s at 55 °C and 1.5 min at 72 °C, 30 cycles of 45 s at 94 °C, 45 s at 52 °C and 1.30 min at 72 °C, a post elongation step of 6 min at 72 °C for *TEF1* (EF1, EF2) and a pre-denaturation for 3 min at 95 °C, 5 cycles of 45 s at 95 °C, 45 s at 58 °C and 2 min at 72 °C, 5 cycles of 45 s at 95 °C, 45 s at 56 °C and 2 min at 72 °C, 30 cycles of 45 s at 95 °C, 45 s at 52 °C and 2 min at 72 °C, and a post elongation step of 8 min at 72 °C for *RPB2* (5F2 and 7cr). The PCR products were visualized by electrophoresis on 1% (w/v) agarose gels. The sequencing PCR was performed as follows: 1 min at 95 °C followed by 30 cycles consisting of 10 s at 95 °C, 5 s at 50 °C and 2 min 60 °C. The reactions were purified with Sephadex G-50 fine (GE Healthcare Bio-Sciences, Uppsala, Sweden), and the sequencing was conducted on an ABI 3730xL automatic sequencer (Applied Biosystems) with a BigDye v3.1 terminator cycle sequencing kit (Applied Biosystems).

### Identification

The strains were identified by BLAST in GenBank, *Fusarium* MLST (http://www.cbs.knaw.nl/fusarium/)^20^ and the FUSARIUM-ID (http://isolate.fusariumdb.org/)^21^ databases. In addition, the phylogenetic placements with species/haplotypes within species complexes were verified with available databases that are specific for *Fusarium*.

### Phylogenetic analyses

Sequences of *TEF1* and *RPB2* were undertaken to extend the genetic characterization of 127 isolates of *Fusarium* species previously characterized in terms of morphological characteristics. The sequences were edited using SeqMan in the Lasergene package (DNAstar, Madison, WI, USA). A phylogenetic approach was used to investigate the relationship between 65 strains of *Fusarium* species including type and reference strains. The sequences were aligned using MAFFT v. 7.127 (http://mafft.cbrc.jp) followed by manual adjustments with MEGA v. 6.2.^22^ A combined alignment was constructed for *RPB2* and *TEF1* for both the reference and test strains. The best-fit model of evolution was determined by MEGA v. 6.2.^22^ A bootstrapped maximum-likelihood analysis was performed using RAxMLVI-HPC v. 7.0.3^23^ as implemented on the Cipres portal (http://www.phylo.org/),^24^ with non-parametric bootstrapping using 1000 replicates. Detailed analyses of medically important strains were compared in relation with their clinical cases. For instance, *F. solani* actually represents a complex (that is, the *F. solani* species complex).

### AFLP

The *Fusarium* strains were subjected to amplified fragment length polymorphism (AFLP) genotyping using a previously described method.^25^ However, for the amplification of the DNA fragments, the selective residues (underlined) of the HpyCH4IV-primer (5′-GAT GAG TCC TGA CTA ATG AG-3′) and MseI-primer (5′-Flu-GTA GAC TGC GTA CCC GTAC-3′ MseI-C-selective primer) were replaced. The amplicons were diluted 20 × with double-distilled H_2_O (ddH_2_O); 1 μL of the diluted amplicon was then added to a mixture of 8.9 μL ddH_2_O and 0.1 μL LIZ600 (Applied Biosystems) followed by a heating step for 1 min at 100 °C and cooling to 4 °C. The AFLP fragment analysis was conducted using an ABI3500xL Genetic Analyzer (Applied Biosystems) according to the manufacturer's instructions. The raw data were then inspected visually after importation into BioNumerics v7.5 (Applied Maths, Sint Martens-Latem, Belgium) and analyzed by an Unweighted Pair Group Method with Arithmetic Mean clustering using the Pearson correlation coefficient. The analysis was restricted to DNA fragments in the range of 40–400 bp. The final AFLP dendrograms were based on the combination of sequencing and the AFLP data of both dendrograms.

### Meta-analysis

The authors analyzed the existing medical literature on human cases of fusariosis from 1958 until December 2015. The authors conducted a systematic literature search using PubMed, and the terms ‘*Fusarium'* and ‘fusariosis' were used for the search and both were also used as MeSH words and free words. Studies were only included that reported data for the individual cases because data provided in aggregate often lacked specific information for individual cases. Only cases with either histologically or culturally proven *Fusarium* infection were included. A total of 388 case reports in ~265 published studies were collected on a worldwide basis. The numbers are approximate because some cases have been used in repeated publications. Only cases with either histologically or culturally proven *Fusarium* infection were included (Supplementary Reference S1).

## RESULTS

### Types of articles

A total of 388 cases of fusariosis from 1958 until December 2015 were used in the literature data analysis. This included articles that were mostly single case reports, two patient cases and a series of cases of fusariosis. The reported cases of fusariosis were identified from all over the world, and particularly from tropical and subtropical countries with a large agrarian population such as Brazil, China, Colombia, India and Mexico. The other areas with frequent fusariosis were Australia, South Africa, Turkey and the Americas. *Fusarium* infections have also been reported from different countries in eastern and western Europe.

#### Patient characteristics

An overview of the cases of fusariosis published in the medical literature, which includes the great majority of cases published to date, is provided in Table 2. The majority of patients were male (*n*=253; 65.2% mean 41 years; range three months–83 years). Over a third of the patients (*n*=143; 36.9%) had various underlying conditions at the time when the *Fusarium* infection was diagnosed. Causes of immunosuppression were hematological diseases and hematologic malignancies (*n*=122; 31.4%) and cancer of the solid organs (*n*=17; 4.8%). Other causes of immunosuppression were medication (*n*=140; 36%), which included antibiotic (*n*=34; 8.8%) and steroid treatment (*n*=10; 2.6%). Pathogen introduction was ranked as trauma (*n*=18; 4.6%), indwelling catheters (*n*=2; 0.5%), nasogastric tubes (*n*=2; 0.5%) and dialysis (*n*=3; 0.77%). No metabolic disorders, such as diabetes, were recorded in association with infection.

### Type of infections

Infections due to *Fusarium* were predominantly found to be superficial and subcutaneous (*n*=174; 44.8%), occurring on the skin (*n*=62; 16%), eyes (*n*=66; 17%) and nails (*n*=25; 6.4%). Deep infections involved bone, joint and lung (*n*=4; 1%), heart (*n*=3; 0.77%), and peritoneum (*n*=2; 0.5%). The sum of the invasive and disseminated cases was *n*=109 (28%), some of which were associated with fungemia (*n*= 25; 6.4%) or disseminated disease with brain abscesses (*n*=4; 1% Table 2).

### Treatment

An overview of the reported treatment of the cases of fusariosis is shown in Table 3. The most widely used antifungal agent was amphotericin B deoxycholate (*n*=198; 51%), followed by liposomal amphotericin B (*n*=45; 11.6%), voriconazole (*n*=42; 10.8%), 5-flucytosine (*n*=30; 7.7%), itraconazole (*n*=26; 6.7%), fluconazole (*n*=25; 6.4%) and ketoconazole (*n*=19; 4.9%).

The antifungal combinations used in treating fusariosis were given either as a two- or a three-drug combination. The most frequently used combination of two drugs was amphotericin B with voriconazole (*n*=24; 6%), followed by amphotericin B with 5-flucytosine (*n*=20; 5%), amphotericin B with ketoconazole (*n*=4; 1%) and amphotericin B with fluconazole (*n*=4; 1%). Other combinations were used in one or two cases. Triple combinations were used in 14 cases (*n*=14; 3.6%). In addition, surgery with antifungal treatment was used in 80 cases (20.6%). In addition to antifungal therapy and surgery, granulocyte transfusions or granulocyte–colony-stimulating factor transfusions were also used. Only seven isolates were associated with cases where no treatment was reported (Table 3). It was not possible to look at the changes in treatment over time, although the authors assume that azole treatments have increased while AmB has declined. With the current guidelines, liposomal amphotericin B (*n*=45; 11.6%) and voriconazole (*n*=42; 10.8%) are very similar according to the data from the reported cases.

### Genetic analysis

**Figure 1:**
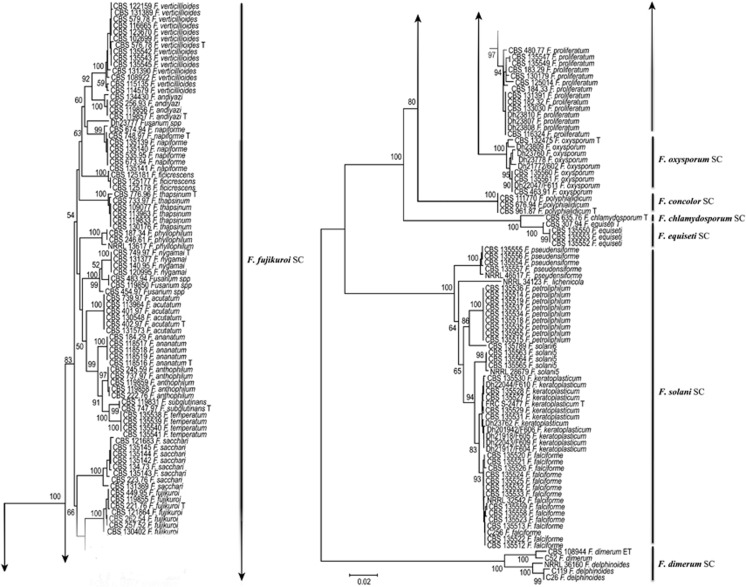
A phylogenetic tree resulting from the RAxML analysis for the *RPB2* and *TEF1* genes. The total alignment length is 1302 bp. A maximum-likelihood analysis was performed using RAxML with non-parametric bootstrapping using 1000 replicates. The numbers above the branches are bootstrap support values ≥0.70. The outgroup was the epitype (ET) strain of *F. dimerum* CBS 108944.

A total of 127 *Fusarium* strains deposited in the CBS-KNAW collection were partially sequenced for *RPB2* and *TEF1*. The resulting two phylogenies yielded almost identical topologies with similar resolution. Almost all of the strains of known species in all complexes of *Fusarium* formed independent clades in each tree. A concatenated tree (Figure 1), including all major human–pathogenic complexes of *Fusarium*, was based on 146 selected sequences. The lengths of the generated sequence data were 795 and 507 bp for *RPB*2 and *TEF1*, respectively. Of the 1302 nucleotides sequenced, 720 (55.1%) were constant, 551 (42.2%) were parsimony informative and 576 (44.1%) were variably and parsimony non-informative using MEGA v. 6.2.^22^ The combined tree was subdivided into several species complexes with high bootstrap values (Figure 1). Seven clades represented human opportunists within the *F. solani* species complex. Thirteen groups represented opportunistic species in the *F. fujikuroi* species complex with smaller human-associated clusters in the FOSC and to a lesser extent in the *F. chlamydosporum*, *F. polyphialidicum* (syn. *F. concolor*), *F. dimerum* and *F. incarnatum* species complexes. Strains CBS 454.97, CBS 483.94 and CBS 119850 were identified morphologically as *F. napiforme* but formed a separate cluster that was different from the three strains including the type strain of *F. napiforme* (Figure 1).

**Figure 2:**
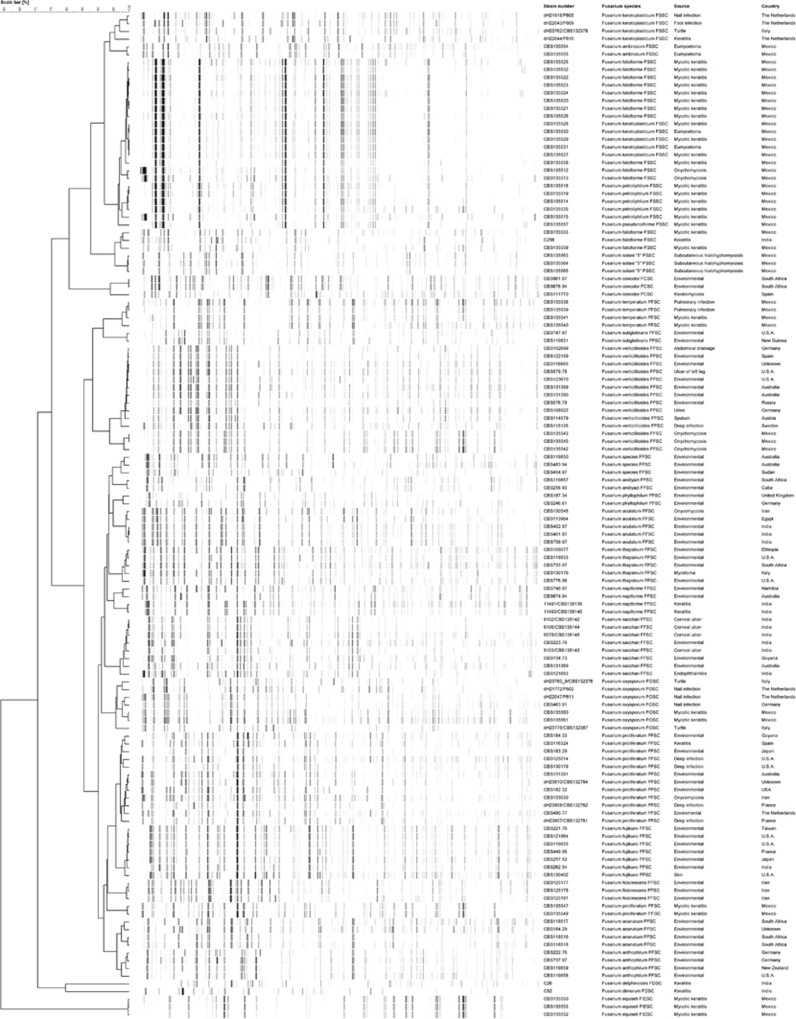
Clustering of the amplified fragment length polymorphism banding pattern of *Fusarium* spp. combined with a sequence analysis of *RPB2* and *TEF1* constructed by Bionumerics v7.5 (Applied Maths). The dendrogram was generated using the Unweighted Pair Group Method with Arithmetic Mean algorithm.

The AFLP profiles contained ~50−60 fragments in the range of 40−400 bp. The AFLP dendrogram comprised seven main clusters at the species complex level and additional subgroups within the main species clusters revealed genetic diversity within each species complex (Figure 2). However, the profiles did not significantly vary between the *F. solani* species complexes, such as *F. falciforme*, *F. keratoplasticum*, *F. lichenicola F. petroliphilum* and *F. pseudensiforme*, whereas there was significant AFLP variation between isolates within the *F. fujikuroi* species complex with separate profiles for each species and within other species complexes of *F. chlamydosporum*, *F. concolor*, *F. dimerum*, *F. incarnatum*-*equiseti* and *F. oxysporum*.

When comparing the AFLP clusters with the distribution of DNA sequence lineages, the groups were largely concordant. Groups 1−7 matched with previous identifications using *RPB2* and *TEF1* sequences. The *Fusarium concolor* species complex had one clinical subgroup, the *F. dimerum* species complex had two and the *Fusarium fujikuroi* species complex consisted of 16 clinical subgroups (15 named subgroups and 1 unnamed molecular lineage). The FIESC had a single clinical group, the FOSC was divided into two subgroups and the *F. solani* species complex comprised six named and one unnamed subgroup. The AFLP clusters and subclusters were almost identical to the sequencing identifications except for few strains within the *F. solani* species complex (Figure 2). The AFLP clusters were based on sequencing and the AFLP data generated from combinations of both dendrograms. In this study, similar clinical presentations of fusariosis were observed among the different AFLP species/genotypes, and there seems to be no relation between species and clinical presentation.

## DISCUSSION

The molecular epidemiology of *Fusarium* was investigated based on the genetic data generated for the *RPB2* and *TEF1* sequence analysis supplemented with the AFLP fingerprint data. The current study provides information on the locality, sources, species and geographic distribution in countries in which these fungi are common such as Brazil, China, Colombia, India, Mexico and the USA. One hundred and thirty *Fusarium* isolates, including eight type strains deposited at the CBS-KNAW collection, were included. Overall, there was a good correlation between sequence analysis and AFLP genotypes. To analyze the genetic relationships among the AFLP and sequencing genotypes, we used phylogenetic algorithms, which are commonly used to detect evidence of population subdivision and differentiation.^26^

The second largest subunit of RNA polymerase (*RPB2*) and *TEF1* showed high resolution with high species-level resolution, distinguishing 29 *Fusarium* species including the most common clinically important *Fusarium* species *F. falciforme*, *F*. *keratoplasticum*, *F. oxysporum*, *F. petroliphilum*, *F. proliferatum* and *F. verticillioides*. Furthermore, these two loci were able to distinguish between other rare *Fusarium* species that cause a variety of infections (Table 1).

Although human opportunists were highlighted in many studies focusing on specific regions of the world and specific types of infections,^10, 11, 12, 13, 27, 28, 29, 30, 31, 32, 33, 34^ the 127 *Fusarium* strains from the current study were collected from 26 countries in six continents and included clinical and environmental strains and isolates from cold blooded animals. Of these, Australia, Brazil, India, Mexico and the USA were among the top 10 countries with the highest *Fusarium* infections based on clinical isolates in the CBS collection. Not surprisingly, 75 of the 127 patients from this study acquired their infection in one of these countries.

Previously, the majority of the clinically relevant *Fusarium* species were classified as two species complexes that in the past were referred to as a single species, *F. oxysporum* and *F. solani*.^35^ Approximately 80% of human infections are caused by members of both species complexes,^36^ but a significant share of infections is caused by the following novel species complex members: *F. dimerum, F. fujikuroi* and *F. incarnatum-equiseti*. Within the *F. solani* complex, there are six recognized species and one unnamed lineage (FSSC6) clinically involved in fusariosis (Figure 1). Of these species, *F. falciforme* (*n*=14/127 cases; 11%) was the dominant species in our study and mainly isolated from keratitis cases in Brazil, India and Mexico. Recently, Hassan *et al.*^13^ showed that the majority of keratitis cases (*n*=46/65 cases; 70.7%) were *F. falciforme*. This species is emerging as one of the most virulent *Fusarium* species associated with fusariosis and keratitis.^15, 36, 37^

In the 2005–2006 mycotic keratitis outbreaks in Southeast Asia and North America that were associated with a contact lens cleaning solution, *F. petroliphilum* and *F. keratoplasticum* were the most common species,^36^ which is consistent with the current study. The AFLP genotypic variability was higher in the environmental species than in the clinical species. A potential explanation is that not all environmental genotypes are sufficiently adapted to the host tissue and are not selected or perhaps a sampling effect is involved. Zhang *et al.*^35^ studied the *F. solani* species complex, specifically those species that cause infections in humans and plants, and concluded that clinical isolates often shared multi-locus haplotypes with isolates from different environmental sources, including hospital locations. An increase of fusariosis among immunosuppressed patients was noted in the bone marrow transplant unit and among patients with superficial infections in a hospital in Rio de Janeiro, Brazil.^38^ These authors concluded that this increase might be due to airborne conidia circulating in this geographical region. Short *et al.*^36^ concluded that there is no evidence that clinical isolates differ from those collected from other sources.

The large diversity of the FOSC is not completely resolved, and it is not yet known whether the species have one or several phylogenetic origins or whether a single species or a species complex is concerned. From a traditional taxonomic point of view, *F. oxysporum* isolates are differentiated from each other based on the pathogenicity as *formae speciales*, but this has been shown to be an unreliable approach.^8^ In addition, the species delimitation was for the FOSC, and at least 26 sequence types within the complex were involved in human infections.^39^ Our FOSC clinical isolates were distributed throughout the complex, although some clustering was found in the clade marked ‘sequence type 33', which is based on *TEF1* alone, and this sequence type is considered the most common clade that contains clinical *F. oxysporum* strains. The remaining species complexes of *F. chlamydosporum*, *F. concolor*, *F. dimerum* and *F. incarnatum-equiseti* form separate clusters in the highly resolved sequence-based maximum likelihood tree (Figure 1).

The FIESC compromises 28 phylogenetically distinct lineages,^34^ and only 2 are named and mainly involved in human infections (*F. incarnatum* and *F. equiseti*).^40^ Although several members of the FIESC were included in the CDC *Fusarium* keratitis outbreak investigations, these species have not yet been observed to occur in epidemics or cause outbreaks. Concerning geography, 51 clinical isolates were recovered from the United States, and this revealed that phylogenetically diverse human opportunists are well represented in North America.^40^ In our study, three clinical *F. equiseti* strains originated from Mexico, and this might suggest that species of this complex are common in this region. The virulence of members of the FIESC has been ascribed to their production of type A and B trichothecene mycotoxins.^39^

*F. dimerum* and *F. delphinoides* belong to the *F. dimerum* species complex, and both were isolated from superficial and disseminated infections.^15^ In our data set, a supported clade of FDSC matching with the AFLP data mainly contained strains from India, and this might suggest a regional prevalence. *F. chlamydosporum* was reported in disseminated infections in patients with aplastic anemia and lymphocytic lymphoma from the United States^41, 42^ CBS 111770 (*F. concolor*) is the only clinical strain in the *F. concolor* species complex, and it was reported in a keratitis case from Spain.^43^

By comparing AFLP and MLST data, *F. falciforme* and *F. keratoplasticum* appear to be widely distributed, at least in Mexico, North America, Europe and India, with dominancy in superficial infections, including keratitis and onychomycosis. *F. petroliphilum* is the second most diverse species and is also frequently involved in disseminated infections. *F. solani sensu stricto*, ‘5', which was recently described as *Fusisporium* (*Fusarium*) *solani* (FSSC5),^7^ contains strains such as CBS 135559, CBS 135564 and CBS 135565, which originate from Mexico, and shows significant occurrence in keratitis cases. This species was also recently reported in Asia (India and Qatar).^13, 16^ Given the large distances of identical strains occurring in many different countries, airborne distribution seems likely. However, the presence of *F. incarnatum, F. equiseti* and *F. chlamydosporum* in clinical samples from various infections in North America remains puzzling but can perhaps be explained by sampling effects.

As previously noted,^44^ the *F. fujikuroi* complex contains the highest number of species. In our study, 15 supported clades were recognized in all of the molecular analyses (Figures 1 and 2). Nearly all of the clades have various geographic distributions. Within the *F. fujikuroi* species complex, *F. proliferatum* and *F. verticillioides* were the dominant clinically relevant species, having a global distribution and dominating in disseminated infections. *F. sacchari* is the second most prevalent species and was often isolated from keratitis restricted to India. Although *F. nygamai* and *F. napiforme* are the most multidrug-resistant species within the *F. fujikuroi* complex,^45^ their presence in human infections is rare. *F. acutatum* was reported from nail infections in four cases in Qatar, showed a low degree of variability and has been suggested to be clonal.^16^ These results emphasize that *F. acutatum* is an emerging human opportunist, which thus far was only detected in Asia. Sequence analysis of *RPB2* and *TEF1,* and AFLP showed that the strains CBS 119850, CBS 483.94 and CBS 454.97 were nested within the *F. fujikuroi* complex and close to *F. nygamai* and *F. andiyazi*, forming a well-supported monophyletic branch suggestive of a novel species.

Deep fusariosis is rare in healthy individuals; a single brain infection has been reported.^46^ Local infections may occur after a direct inoculation or tissue breakdown by trauma or the entrance of foreign bodies. The treatment of superficial infections is usually successful and requires surgery, the removal of the foreign body and antifungal therapy. The most important risk factors for severe fusariosis are prolonged neutropenia and T-cell immunodeficiency in patients suffering from a hematologic malignancy. *Fusarium* infections in the majority of these cases were due to neutropenia. Furthermore, in solid organ transplant recipients and cancer patients with neutropenia, infections due to *Fusarium* spp. increased and led to disseminated infection. Patients develop painful skin lesions, which vary from papules to nodules with or without central necrosis.^47^ In the majority of disseminated infections, secondary skin lesions led to a diagnosis in >50% of the patients and preceded fungemia by ~5 days.^48^ In contrast to aspergillosis, fusariosis frequently shows positive blood cultures because *Fusarium* conidia are hydrophilic and allow dissemination.^47^ Comparing fusariosis with mucormycosis,^49^ solid tumors and diabetes do not seem to be important risk factors. Only 17 (4.8%) cases were found in patients with solid tumors, and seven infections were reported in patients with diabetes mellitus. No underlying conditions were observed in 20 (5%) of the cases.

*Fusarium* treatment depends on the site of infection. Surgery with antifungals was used in 80 cases (20.6%). Disseminated fusariosis in immunocompromised patients is usually treated with amphotericin B and voriconazole as the first-line therapy, which is suggested by recent guidelines.^50^ In our literature review, most antifungal therapy was amphotericin B deoxycholate, followed by liposomal amphotericin B and voriconazole. The most commonly used combination is amphotericin B/voriconazole followed by amphotericin B/5-flucytosine. Triple combinations were used in 14 cases with different antifungals.

The major findings of the present study include the following: (i) human-associated fusaria were nested within seven species complexes (that is, *F. chlamydosporum*, *F. concolor*, *F. dimerum*, *F. fujikuroi*, *F. incarnatum-equiseti*, *F. oxysporum* and *F. solani*), (ii) the three most common species presented in both the clinical and environmental groups are *F. falciforme* and *F. keratoplasticum* (members of *F. solani* species complex) followed by *F. oxysporum* (FOSC), (iii) most of the reported *Fusarium* species in this study were shared among the patients and the environment, and this might be due to the colonization of some patients with *Fusarium* isolates from the environment; hence, there is genetic similarity between the clinical and environmental isolates of the same *Fusarium* species, and (iv) the species distributions show some evidence of geographical clustering among some of the species studied, although the present study is limited by an over-representation of isolates from Mexico and India.

## Figures and Tables

**Table 1:** Isolates of *Fusarium* included in this study that were used for the sequence analysis and amplified fragment length polymorphism analysis, except for six *RPB2* and *TEF1*, which were retrieved from GenBank

**CBS number**	**Species name**	**Country**	**Source**	**GenBank accession number**	
				**TEF1**	**RPB2**
CBS 130548	*F. acutatum*	Iran	Onychomycosis (Human)	KR071756	KU604289
CBS 113964	*F. acutatum*	Egypt	Environmental	KR071759	KU604290
CBS 739.97	*F. acutatum*	India	Environmental	KR071757	KU604288
CBS 401.97	*F. acutatum*	India	Environmental	KR071755	KU604287
CBS 402.97	*F. acutatum*	India	Environmental	KR071754	KT154005
CBS 118517	*F. ananatum*	South Africa	Environmental	KR071761	KU604273
CBS 118518	*F. ananatum*	South Africa	Environmental	KU711690	KU604271
CBS 118516	*F. ananatum*	South Africa	Environmental	KR071760	KU604269
CBS 184.29	*F. ananatum*	Unknown	Environmental	KR071762	KU604272
CBS 256.93	*F. andiyazi*	Cuba	Environmental	KR071719	KU604231
CBS 119857	*F. andiyazi*	South Africa	Environmental	KP662901	KT154004
CBS 737.97	*F. anthophilum*	Germany	Environmental	KU711685	KU604277
CBS 222.76	*F. anthophilum*	Germany	Environmental	KR071766	KT154006
CBS 119858	*F. anthophilum*	USA	Environmental	KR071764	KU604275
CBS 119859	*F. anthophilum*	New Zealand	Environmental	KR071765	KU604279
CBS 961.87	*F. concolor*	South Africa	Environmental	KR071773	KU604556
CBS 676.94	*F. concolor*	South Africa	Environmental	KR071774	KU604237
CBS 111770	*F. concolor*	Spain	Keratitis (Human)	KU711719	KU604323
C26	*F. delphinoides*	India	Keratitis (Human)	KU711775	KU604380
C52	*F. dimerum*	India	Keratitis (Human)	KU711776	KU604381
CBS 135550	*F. equiseti*	Mexico	Keratitis (Human)	KU711721	KU604324
CBS 135552	*F. equiseti*	Mexico	Keratitis (Human)	KU711723	KU604325
CBS 135553	*F. equiseti*	Mexico	Keratitis (Human)	KU711722	KU604326
CBS 135532	*F. falciforme*	Mexico	Keratitis (Human)	KU711737	KU604356
CBS 135533	*F. falciforme*	Mexico	Keratitis (Human)	KU711738	KU604362
CBS 135521	*F. falciforme*	Mexico	Keratitis (Human)	KU711733	KU604357
CBS 135520	*F. falciforme*	Mexico	Keratitis (Human)	KU711732	KU604363
CBS 135526	*F. falciforme*	Mexico	Keratitis (Human)	KU711734	KU604366
CBS 135524	*F. falciforme*	Mexico	Keratitis (Human)	KU711730	KU604358
CBS 135525	*F. falciforme*	Mexico	Keratitis (Human)	KU711731	KU604359
CBS 135558	*F. falciforme*	Mexico	Keratitis (Human)	KU711736	KU604368
CBS 135559	*F. falciforme*	Mexico	Keratitis (Human)	KU711735	KU604369
CBS 135513	*F. falciforme*	Mexico	Onychomycosis (Human)	KU711724	KU604360
CBS 135512	*F. falciforme*	Mexico	Onychomycosis (Human)	KM401894	KM401892
C256	*F. falciforme*	India	Keratitis (Human)	KU711725	KU604361
CBS 135522	*F. falciforme*	Mexico	Keratitis (Human)	KU711726	KU604364
CBS 135523	*F. falciforme*	Mexico	Keratitis (Human)	KU711727	KU604367
CBS 125177	*F. ficicrescens*	Iran	Environmental	KP662898	KT154001
CBS 125178	*F. ficicrescens*	Iran	Environmental	KP662899	KT154002
CBS 125181	*F. ficicrescens*	Iran	Environmental	KP662900	KT154003
CBS 449.95	*F. fujikuroi*	France	Environmental	KR071742	KU604259
CBS 257.52	*F. fujikuroi*	Japan	Environmental	KU711678	KU604257
CBS 262.54	*F. fujikuroi*	India	Environmental	KR071744	KU604256
CBS 221.76	*F. fujikuroi*	Taiwan	Environmental	KR071741	KU604255
CBS 130402	*F. fujikuroi*	USA	Human skin (Human)	KU711677	KU604261
CBS 121864	*F. fujikuroi*	USA	Environmental	KR071743	KU604258
CBS 119855	*F. fujikuroi*	USA	Environmental	KU711679	KU604260
CBS 454.97	*Fusarium* sp	Sudan	Environmental	KU711697	KU604266
CBS 483.94	*Fusarium* sp	Australia	Environmental	KU711698	KU604267
CBS 119850	*Fusarium* sp	Australia	Environmental	KU711699	KU604268
CBS 135528	*F. keratoplasticum*	Mexico	Keratitis (Human)	KU711743	KU604338
dH22044/F610	*F. keratoplasticum*	Netherlands	Keratitis (Human)	KU711744	KU604339
CBS 135527	*F. keratoplasticum*	Mexico	Keratitis (Human)	KU711742	KU604340
CBS 135531	*F. keratoplasticum*	Mexico	Eumycetoma (Human)	KU711741	KU604353
CBS 135530	*F. keratoplasticum*	Mexico	Eumycetoma (Human)	KU711740	KU604352
CBS 135529	*F. keratoplasticum*	Mexico	Keratitis (Human)	KU711739	KU604354
					
dH21918/F605	*F. keratoplasticum*	Netherlands	Nail infection (Human)	KU711746	KU604344
dH22043/F609	*F. keratoplasticum*	Netherlands	Foot infection (Human)	KU711747	KU604341
CBS 748.97	*F. napiforme*	Namibia	Environmental	KR071712	KU604233
CBS 674.94	*F. napiforme*	Australia	Environmental	KR071713	KU604239
CBS 135139	*F. napiforme*	India	Keratitis (Human)	KR071717	KU604234
CBS 135140	*F. napiforme*	India	Keratitis (Human)	KR071714	KU604235
dH 21772/F602	*F. oxysporum*	Netherlands	Nail infection (Human)	KU711714	KU604318
dH22047/F611	*F. oxysporum*	Netherlands	Nail infection (Human)	KU711711	KU604314
CBS 135560	*F. oxysporum*	Mexico	Keratitis (Human)	KU711709	KU604317
CBS 135561	*F. oxysporum*	Mexico	Keratitis (Human)	KU711710	KU604316
CBS 463.91	*F. oxysporum*	Germany	Nail infections (Human)	KU711712	KU604315
CBS 135515	*F. petroliphilum*	Mexico	Keratitis (Human)	KU711760	KU604336
CBS 135518	*F. petroliphilum*	Mexico	Keratitis (Human)	KU711762	KU604334
CBS 135519	*F. petroliphilum*	Mexico	Keratitis (Human)	KU711765	KU604331
CBS 135535	*F. petroliphilum*	Mexico	Keratitis (Human)	KU711761	KU604335
CBS 135514	*F. petroliphilum*	Mexico	Mycotic keratitis (Human)	KU711767	KU604330
CBS 187.34	*F. phyllophilum*	UK	Environmental	KU711680	KU604300
CBS 246.61	*F. phyllophilum*	Germany	Environmental	KU711681	KU604301
CBS 480.77	*F. proliferatum*	Netherlands	Environmental	KR071736	KU604245
CBS 182.32	*F. proliferatum*	USA	Environmental	KR071734	KU604246
CBS 183.29	*F. proliferatum*	Japan	Environmental	KR071735	KU604242
CBS 184.33	*F. proliferatum*	Guyana	Environmental	KR071737	KU604244
CBS 125014	*F. proliferatum*	USA	Deep infection (Human)	KR071738	KU604243
CBS 131391	*F. proliferatum*	Australia	Environmental	KR071732	KU604247
CBS 133030	*F. proliferatum*	Iran	Onycomycosis (Human)	KR071733	KU604248
CBS 135547	*F. proliferatum*	Mexico	Keratitis (Human)	KU711675	KU604254
CBS 135549	*F. proliferatum*	Mexico	Keratitis (Human)	KU711676	KU604253
CBS 116324	*F. proliferatum*	Spain	Keratitis (Human)	KR071731	KU604249
CBS 130179	*F. proliferatum*	USA	Deep infection (Human)	KR071739	KU604241
dH23807/CBS 132761	*F. proliferatum*	France	Deep infection (Human)	KU711673	KU604250
dH23808/CBS 132762	*F. proliferatum*	France	Deep infection (Human)	KU711674	KU604252
dH23810/CBS 132764	*F. proliferatum*	Unknown	Environmental	KU711672	KU604251
CBS 135554	*F. pseudensiforme*	Mexico	Eumycetoma (Human)	KU711769	KU604377
CBS 135555	*F. pseudensiforme*	Mexico	Eumycetoma (Human)	KU711770	KU604375
CBS 135557	*F. pseudensiforme*	Mexico	Keratitis (Human)	KU711771	KU604376
CBS 135142	*F. sacchari*	India	Corneal ulcer (Human)	KR071749	KU604304
CBS 135143	*F. sacchari*	India	Corneal ulcer (Human)	KR071748	KU604307
CBS 135144	*F. sacchari*	India	Corneal ulcer (Human)	KR071750	KU604305
CBS 135145	*F. sacchari*	India	Corneal ulcer (Human)	KR071751	KU604306
CBS 223.76	*F. sacchari*	India	Environmental	KU711669	KU604309
CBS 134.73	*F. sacchari*	Guyana	Environmental	KR071753	KU604303
CBS 131369	*F. sacchari*	Australia	Environmental	KR071752	KU604302
CBS 121683	*F. sacchari*	India	Endophthalmitis (Human)	KR071747	KU604308
CBS 135563	*F. solani* (FSSC5)	Mexico	Hyalohyphomycosis (Human)	KU711758	KU604372
CBS 135564	*F. solani* (FSSC5)	Mexico	Hyalohyphomycosis (Human)	KU711759	KU604373
CBS 135565	*F. solani* (FSSC5)	Mexico	Hyalohyphomycosis	KU711757	KU604371
CBS 119831	*F. subglutinans*	New Guinea	Environmental	KR071769	KU604281
CBS 747.97	*F. subglutinans*	USA	Environmental	KU711691	KU604280
CBS 135538	*F. temperatum*	Mexico	Pulmonary infection (Human)	KF956082	KU604283
CBS 135539	*F. temperatum*	Mexico	Pulmonary infection (Human)	KF956083	KU604286
CBS 135540	*F. temperatum*	Mexico	Keratitis (Human)	KF956084	KU604285
CBS 135541	*F. temperatum*	Mexico	Keratitis (Human)	KF956085	KU604284
CBS 776.96	*F. thapsinum*	USA	Environmental	KR071726	KU604294
CBS 733.97	*F. thapsinum*	South Africa	Environmental	KR071730	KU604299
CBS 130176	*F. thapsinum*	Italy	Human mycetoma (Human)	KR071727	KU604298
CBS 119833	*F. thapsinum*	USA	Environmental	KU711717	KU604297
CBS 109077	*F. thapsinum*	Ethiopia	Environmental	KR071728	KU604295
CBS 114579	*F. verticillioides*	Austria	Sputum (Human)	KU711696	KU604220
CBS 115135	*F. verticillioides*	Sweden	Deep infection (Human)	KR071710	KU604217
CBS 131390	*F. verticillioides*	Australia	Environmental	KR071711	KU604225
CBS 116665	*F. verticillioides*	Unknown	Environmental	KR071705	KU604221
CBS 135542	*F. verticillioides*	Mexico	Onychomycosis (Human)	KU711693	KU604227
CBS 135543	*F. verticillioides*	Mexico	Onychomycosis (Human)	KU711694	KU604228
CBS 135545	*F. verticillioides*	Mexico	Onychomycosis (Human)	KX584417	KU604229
CBS 576.78	*F. verticillioides*	Russia	Environmental	KR071703	KU604216
CBS 579.78	*F. verticillioides*	USA	Ulcer of left leg (Human)	KR071706	KU604223
CBS 122159	*F. verticillioides*	Spain	Environmental	KR071707	KU604224
CBS 123670	*F. verticillioides*	USA	Environmental	KR071708	KU604222
CBS 102699	*F. verticillioides*	Germany	Abdominal drainage (Human)	KR071704	KU604218
CBS 108922	*F. verticillioides*	Germany	Urine (Human)	KR071709	KU604219
CBS 131389	*F. verticillioides*	Australia	Environmental	KU711695	KU604226

**Table 2:** Characteristics of 388 patients with fusariosis and literature cases from 1958 until 2015

**Characteristic**	**Number of patients**
Total	388
Age, years (range)	3 months−82 years
Sex, M:F:unknown	253 (65.3%):125 (32.2%):10 (2.5%)

*Underlying condition*	
Transplantation	
Liver	5 (1.2%)
Lung	4 (1%)
Bone morrow	29 (7.5%)
Multivisceral (stomach, duodenum, pancreas and intestine)	1 (0.25%) 7 (1.8%)
Kidney	3 (0.77%)
Heart	4 (1%)
Stem cells	38 (9.8%)
Trauma/burns	27 (7%)
Foreign body	18 (4.6%)
Contact lens	4 (1%)
Catheter	2 (0.5%)
Graft	3 (0.77%)
Nasogastric tube	3 (0.77%)
Dialysis	4 (1%)
Cancer	
Hematologic	122 (31.4%)
Solid organ	17 (4.8%)
Medication	
Antibiotics	140 (36%)
Steroids	34 (8.8%)
No	20 (5%)
	
*Site of infection*	
Superficial	
Skin	62 (16%)
Eye	66 (17%)
Nail	25 (6.44)
Bone	4 (1%)
Joint	4 (1%)
Lung	4 (1%)
Endocarditis	3 (0.77%)
Peritoneum	2 (0.5%)
Perinephric abscess	2 (0.5%)
Disseminated	109 (28%)
Blood	25 (6.4%)
Brain	4 (1%)

Abbreviations: female, F; male, M.

**Table 3:** Treatment administered to 388 patients with fusariosis

*Treatment*	
*Primary treatment (one drug)*	
Amphotericin B	
Deoxycholate	198 (51%)
Lipid/liposomal	45 (11.6%)
Voriconazole	42 (10.8)
Flucytosine	30 (7.7%)
Itraconazole	26 (6.7%)
Fluconazole	25 (6.4%)
Ketoconazole	19 (4.9%)
Rifampicin	13 (3.4)
Posaconazole	3 (0.77%)
Terbinafine	—
Natamycin	2 (0.5%)
Surgery	80 (20.6%)
G-CSF	25 (6.4%)
G-Transfusion	13 (3.4%)
No therapy	7 (1.8%)

* Combinations (two drugs)*	
Amphotericin B/voriconazole	24 (6%)
Amphotericin B/5-flucytosine	20 (5%)
Amphotericin B/ketoconazole	4 (1%)
Amphotericin B/fluconazole	4 (1%)
Amphotericin B/posaconazole	2 (0.5%)
Amphotericin B/itraconazole	2 (0.5%)
Amphotericin B/caspofungin	1 (0.26%)
Voriconazole/caspofungin	2 (0.5%)
Voriconazole/anidulafungin	1 (0.26%)
Voriconazole/terbinafine	1 (0.26%)
Ketoconazole/terbinafine	1 (0.26%)
Ketoconazole/rifampicin	1 (0.26%)
Itraconazole/terbinafine	2 (0.5%)
Itraconazole/terbinafine	1 (0.26%)

* Combinations (three drugs)*	
Amphotericin B/flucytosine/rifampicin	2 (0.5%)
Amphotericin B/itraconazole/rifampicin	2 (0.5%)
Amphotericin B/flucytosine/ketoconazole	2 (0.5%)
Amphotericin B/fluconazole/voriconazole	2 (0.5%)
Amphotericin B/fluconazole/itraconazole	1 (0.26%)
Amphotericin B/itraconazole/voriconazole	1 (0.26%)
Amphotericin B/flucytosine/itraconazole	1 (0.26%)
Amphotericin B/fluconazole/rifampicin	1 (0.26%)
Amphotericin B/voriconazole/micafungin	1 (0.26%)
Amphotericin B/voriconazole/terbinafine	1 (0.26%)

Abbreviation: granulocyte–colony-stimulating factor, G-CSF.

## Supplementary Material

Supplementary Reference S1
