# Supplementary material for: Global molecular epidemiology and genetic diversity of Fusarium, a significant emerging group of human opportunists from 1958 to 2015
Source: Emerg Microbes Infect. 2016 Dec 7;5(12):e124–. doi: 10.1038/emi.2016.126 (PMC5180370; doi:10.1038/emi.2016.126)
Supplement: Supplementary Reference S1 [file emi2016126x1.docx]

**Supplementary Reference S1**

1. Mikami R, Stemmermann GN. Keratomycosis caused by *Fusarium oxysporum*. Am J Clin Pathol. 1958; 29 (3): 257-62,
2. Ritchie EB, Pinkerton ME. *Fusarium oxysporum* infection of the nail; report of cases. AMA Arch Derm. 1959; 79 (6):705-8.
3. Lynn JR. *Fusarium* Keratitis Treated with Cycloheximide. Am J Ophthalmol. 1964;58: 637-41.
4. Benjamin RP, Callaway L, Conant NF. Facial granuloma associated with *Fusarium* infection. Arch Dermatol. 1970;101 (5):598-600.
5. Cho CT, Vats TS, Lowman JT, Brandsberg JW, Tosh FE. *Fusarium solani* infection during treatment for acute leukemia. J Pediatr. 1973; 83 (6):1028-31.
6. Abramowsky CR, Quinn D, Bradford WD, Conant NF. Systemic infection by *Fusarium* in a burned child. The emergence of a saprophytic strain. J Pediatr. 1974;84 (4):561-4.
7. Gutmann L, Chou SM, Pore RS. Fusariosis, myasthenic syndrome, and aplastic anemia. Neurology. 1975; 25 (10): 922-6.
8. Zapater RC, Arrechea A. Mycotic keratitis by *Fusarium*. A review and report of two cases. Ophthalmologica. 1975; 170 (1): 1-12.
9. Bourguignon RL, Walsh AF, Flynn JC, Baro C, Spinos E. *Fusarium* species osteomyelitis. Case report. J Bone Joint Surg Am. 1976;58 (5):722-3.
10. Young NA, Kwon-Chung KJ, Kubota TT, Jennings AE, Fisher RI. Disseminated infection by *Fusarium moniliforme* during treatment for malignant lymphoma. J Clin Microbiol. 1978; 7(6): 589-94,.
11. Lieberman TW, Ferry AP, Bottone EJ. *Fusarium solani* endophthalmitis without primary corneal involvement. Am J Ophthalmol. 1979;88 (4): 764-7.
12. Rowsey JJ, Acers TE, Smith DL, Mohr JA, Newsom DL, Rodriguez J. *Fusarium oxysporum* endophthalmitis. Arch Ophthalmol. 1979;97(1): 103-5.
13. DiSalvo AF, Fickling AM. A case of nondermatophytic toe onychomycosis caused by *Fusarium oxysporum*. Arch Dermatol. 1980;116 (6): 699-700.
14. Mutton KJ, Lucas TJ, Harkness JL. Disseminated *Fusarium* infection. Med J Aust. 1980; 2(11): 624-5.
15. van Dijk E, van den Berg WH, Landwehr AJ. *Fusarium solani* infection of a hypertensive leg ulcer in a diabetic. Mykosen. 1980; 23 (11): 603-6.
16. Singh H, Jamal F, Marahakim MN, Song CC. *Fusarium solani* keratitis. First report from Malaysia. Med J Malaysia. 1981;36 (2): 89-91.
17. Oji EO, Steele DM. *Fusarium solani* keratitis. East Afr Med J. 1982;59 (9): 632-8.
18. Page JC, Friedlander G, Dockery GL. Postoperative *Fusarium* osteomyelitis. J Foot Surg. 1982; 21 (3): 174-6.
19. Jakle C, Leek JC, Olson DA, Robbins DL. Septic arthritis due to *Fusarium solani*. J Rheumatol. 1983; 10 (1): 151-3.
20. Blazar BR, Hurd DD, Snover DC, Alexander JW, McGlave PB. Invasive *Fusarium* infections in bone marrow transplant recipients. Am J Med 1984; 77(4): 645-51.
21. Chaulk CP, Smith RW, Feagler JR, Verdirame J, Commers JR. Fungemia due to *Fusarium solani* in an immunocompromised child. Pediatr Infect Dis. 1986;5 (3): 363-6.
22. June CH, Beatty PG, Shulman HM, Rinaldi MG. Disseminated *Fusarium moniliforme* infection after allogeneic marrow transplantation. South Med J. 1986;79 (4): 513-5.
23. Matsuda T, Matsumoto T. Disseminated hyalohyphomycosis in a leukemic patient. Arch Dermatol. 1986;122 (10):1171-5.
24. Valenstein P, Schell WA. Primary intranasal *Fusarium* infection. Potential for confusion with rhinocerebral zygomycosis. Arch Pathol Lab Med. 1986;110 (8): 751-4.
25. Willemsen MJ, De Coninck AL, Coremans-Pelseneer JE, Marichal- Pipeleers MA, Roseeuw DI. Parasitic invasion of *Fusarium oxysporum* in an arterial ulcer in an otherwise healthy patient. Mykosen. 1986; 29 (6): 248-52.
26. Anaissie E, Kantarjian H, Jones P. Fungal infection caused by *Fusarium* in cancer patients. Am J Clin Oncol. 1987.10 (1): 86-8.
27. Ooi SP, Chen TT, Huang TH, Chang HS, Hsieh HY. Granuloma annularelike skin lesion due to *Fusarium roseum*: therapy with ketoconazole. Arch Dermatol. 1987.123 (2): 167-8.
28. Merz WG, Karp JE, Hoagland M, Jett-Goheen M, Junkins JM, Hood AF. Diagnosis and successful treatment of fusariosis in the compromised host. J Infect Dis. 1988;158 (5): 1046-55.
29. Mowbray DN, Paller AS, Nelson PE, Kaplan RL. Disseminated *Fusarium solani* infection with cutaneous nodules in a bone marrow transplant patient. Int J Dermatol. 1988; 27 (10): 698-701.
30. Nuovo MA, Simmonds JE, Chacho MS, McKitrick JC. *Fusarium solani* osteomyelitis with probable nosocomial spread. Am J Clin Pathol. 1988; 90 (6): 738-41.
31. Richardson SE, Bannatyne RM, Summerbell RC, Milliken J, Gold R, Weitzman SS. Disseminated fusarial infection in the immunocompromised host. Rev Infect Dis. 1988;10 (6): 1171-81.
32. Rippon JW, Larson RA, Rosenthal DM, Clayman J. Disseminated cutaneous and peritoneal hyalohyphomycosis caused by *Fusarium* species: three cases and review of the literature. Mycopathologia. 1988;101 (2):105-11.
33. Summerbell RC, Richardson SE, Kane J. *Fusarium proliferatum* as an agent of disseminated infection in an immunosuppressed patient. J Clin Microbiol. 1988; 26 (1): 82-7.
34. Hemo I, Pe'er J, Polacheck I. *Fusarium oxysporum* keratitis. Ophthalmologica. 1989;198 (1): 3-7.
35. Minor RL Jr, Pfaller MA, Gingrich RD, Burns LJ. Disseminated *Fusarium* infections in patients following bone marrow transplantation. Bone Marrow Transplant. 1989;4 (6): 653-8.
36. Sturm AW, Grave W, Kwee WS. Disseminated *Fusarium oxysporum* infection in patient with heatstroke. Lancet. 1989;1 (8644): 968.
37. Barrios NJ, Kirkpatrick DV, Murciano A, Stine K, Van Dyke RB, Humbert JR. Successful treatment of disseminated *Fusarium* infection in an immunocompromised child. Am J Pediatr Hematol Oncol. 1990;12 (3): 319-24.
38. Gradon JD, Lerman A, Lutwick LI. Septic arthritis due to *Fusarium moniliforme*. Rev Infect Dis. 1990; 12 (4): 716-7.
39. Jorens PG, Van Den Heuvel PA, Van Cauwelaert PA, Parizel GA, Mertens AN. *Fusarium* endocarditis involving aortic valve following coronary artery surgery. Eur Heart J. 1990;11 (5): 476-8.
40. Lupinetti FM, Giller RH, Trigg ME. Operative treatment of *Fusarium* fungal infection of the lung. Ann Thorac Surg. 1990; 49 (6): 991-2.
41. Nadler JP. Disseminated fusarial infection. Rev Infect Dis. 1990; 12 (1): 162.
42. Viscoli C, Castagnola E, Moroni C, Garaventa A, Manno G, Savioli C. Infection with *Fusarium* species in two children with neuroblastoma. Eur J Clin Microbiol Infect Dis. 1990; 9 (10): 773-6.
43. Agamanolis DP, Kalwinsky DK, Krill CE Jr, Dasu S, Halasa B, Galloway PG. *Fusarium* meningoencephalitis in a child with acute leukemia. Neuropediatrics. 1991; 22 (2): 110-2.
44. Robertson MJ, Socinski MA, Soiffer RJ, Finberg RW, Wilson C, Anderson KC, et al. Successful treatment of disseminated *Fusarium* infection after autologous bone marrow transplantation for acute myeloid leukemia. Bone Marrow Transplant. 1991; 8 (2): 143-5.
45. Sayama K, Ohtsuka H, Shiraishi S, Miki Y, Tada M, Matsumoto T. Squamous cell carcinoma arising in long-standing granulomatous hyalohyphomycosis caused by *Fusarium solani*. Arch Dermatol. 1991; 127 (11): 1735-7.
46. Viviani MA, Cofrancesco E, Boschetti C, Tortorano AM, Cortellaro M. Eradication of *Fusarium* infection in a leukopenic patient treated with liposomal amphotericin B. Mycoses. 1991; 34 (5-6): 255-6.
47. Alvarez-Franco M, Reyes-Mugica M, Paller AS. Cutaneous *Fusarium* infection in an adolescent with acute leukemia. Pediatr Dermatol. 1992; 9 (1): 62-5.
48. Brint JM, Flynn PM, Pearson TA, Pui CH. Disseminated fusariosis involving bone in an adolescent with leukemia. Pediatr Infect Dis J. 1992; 11 (11): 965-8.
49. Girmenia C, Arcese W, Micozzi A, Martino P, Bianco P, Morace G. Onychomycosis as a possible origin of disseminated *Fusarium solani* infection in a patient with severe aplastic anemia. Clin Infect Dis. 1992; 14 (5): 1167.
50. Kurien M, Anandi V, Raman R, Brahmadathan KN. Maxillary sinus fusariosis in immunocompetent hosts. J Laryngol Otol. 1992; 106 (8): 733-6.
51. Madhavan M, Ratnakar C, Veliath AJ, Kanungo R, Smile SR, Bhat S. Primary disseminated fusarial infection. Postgrad Med J. 1992; 68 (796): 143-4.
52. Neumeister B, Bartmann P, Gaedicke G, Marre R. A fatal infection due to *Fusarium oxysporum* in a child with Wilms' tumour. Case report and review of the literature. Mycoses. 1992; 35 (5-6): 115-9.
53. Nucci M, Spector N, Lucena S, Bacha PC, Pulcheri W, Lamosa A, et al. Three cases of infection with *Fusarium* species in neutropenic patients. Eur J Clin Microbiol Infect Dis. 1992; 11 (12): 1160-2.
54. Ammari LK, Puck JM, McGowan KL. Catheter-related *Fusarium solani* fungemia and pulmonary infection in a patient with leukemia in remission. Clin Infect Dis. 1993; 16 (1): 148-50.
55. Castagnola E, Garaventa A, Conte M, Barretta A, Faggi E, Viscoli C. Survival after fungemia due to *Fusarium moniliforme* in a child with neuroblastoma. Eur J Clin Microbiol Infect Dis. 1993; 12 (4): 308-9.
56. Caux F, Aractingi S, Baurmann H, Reygagne P, Dombret H, Romand S, et al. *Fusarium solani* cutaneous infection in a neutropenic patient. Dermatology. 1993; 186 (3): 232-5.
57. Engelhard D, Eldor A, Polacheck I, Hardan I, Ben-Yehuda D, Amselem S, et al. Disseminated visceral fusariosis treated with amphotericin B-phospholipid complex. Leuk Lymphoma. 1993; 9 (4-5): 385-92.
58. Melcher GP, McGough DA, Fothergill AW, Norris C, Rinaldi MG. Disseminated hyalohyphomycosis caused by a novel human pathogen, *Fusarium napiforme*. J Clin Microbiol. 1993; 31 (6): 1461-7.
59. Spielberger RT, Falleroni MJ, Coene AJ, Larson RA. Concomitant amphotericin B therapy, granulocyte transfusions, and GM-CSF administration for disseminated infection with *Fusarium* in a granulocytopenic patient. Clin Infect Dis. 1993; 16 (4): 528-30.
60. Wickern GM. *Fusarium* allergic fungal sinusitis. J Allergy Clin Immunol. 1993; 92 (4): 624-5.
61. Ellis ME, Clink H, Younge D, Hainau B. Successful combined surgical and medical treatment of *Fusarium* infection after bone marrow transplantation. Scand J Infect Dis. 1994; 26 (2): 225-8.
62. Hsu CM, Lee PI, Chen JM, Huang LM, Wu MH, Chiu IS, et al. Fatal *Fusarium* endocarditis complicated by hemolytic anemia and thrombocytopenia in an infant. Pediatr Infect Dis J. 1994;13 (12): 1146-8.
63. Louie T, el Baba F, Shulman M, Jimenez-Lucho V. Endogenous endophthalmitis due to *Fusarium*: case report and review. Clin Infect Dis. 1994; 18 (4): 585-8.
64. Rabodonirina M, Piens MA, Monier MF, Gueho E, Fiere D, Mojon M. *Fusarium* infections in immunocompromised patients: case reports and literature review. Eur J Clin Microbiol Infect Dis. 1994;13 (2): 152-61.
65. Freidank H. Hyalohyphomycoses due to *Fusarium* spp. two case reports and review of the literature. Mycoses. 1995; 38 (1-2): 69-74.
66. Leu HS, Lee AY, Kuo TT. Recurrence of *Fusarium solani* abscess formation in an otherwise healthy patient. Infection. 1995; 23 (5): 303-5.
67. Mohammedi I, Gachot B, Grossin M, Marche C, Wolff M, Vachon F. Overwhelming myocarditis due to *Fusarium oxysporum* following bone marrow transplantation. Scand J Infect Dis. 1995; 27 (6): 643-4.
68. Myoken Y, Sugata T, Kyo T, Fujihara M. Oral *Fusarium* infection in a granulocytopenic patient with acute myelogenous leukemia: a case report. J Oral Pathol Med. 1995; 24 (5): 237-40.
69. Raad I, Hachem R. Treatment of central venous catheter-related fungemia due to *Fusarium oxysporum*. Clin Infect Dis. 1995; 20 (3): 709-11.
70. Velasco E, Martins CA, Nucci M. Successful treatment of catheter-related fusarial infection in immunocompromised children. Eur J Clin Microbiol Infect Dis 1995; 14 (8): 697-9.
71. Wolff MA, Ramphal R. Use of amphotericin B lipid complex for treatment of disseminated cutaneous *Fusarium* infection in a neutropenic patient. Clin Infect Dis 1995; 20 (6): 1568-9.
72. Arrese JE, Pierard-Franchimont C, Pierard GE. Fatal hyalohyphomycosis following *Fusarium* onychomycosis in an immunocompromised patient. Am J Dermatopathol 1996; 18 (2): 196-8.
73. Bleggi-Torres LF, de Medeiros BC, Neto JZ, Loddo G, Telles FQ, de Medeiros CR, et al. Disseminated *Fusarium* sp. infection affecting the brain of a child after bone marrow transplantation. Bone Marrow Transplant. 1996;18 (5): 1013-5.
74. Patterson TS, Barton LL, Shehab ZM, Hutter JJ. Amphotericin B lipid complex treatment of a leukemic child with disseminated *Fusarium solani* infection. Clin Pediatr (Phila). 1996; 35 (5): 257-60.
75. Repiso T, Garcia-Patos V, Martin N, Creus M, Bastida P, Castells A. Disseminated fusariosis. Pediatr Dermatol .1996; 13 (2): 118-21.
76. Rombaux P, Eloy P, Bertrand B, Delos M, Doyen C. Lethal disseminated *Fusarium* infection with sinus involvement in the immunocompromised host: case report and review of the literature. Rhinology. 1996;34 (4): 237-41.
77. Arney KL, Tiernan R, Judson MA. Primary pulmonary involvement of *Fusarium solani* in a lung transplant recipient. Chest. 1997;112 (4): 1128-30.
78. Baran R, Tosti A, Piraccini BM. Uncommon clinical patterns of *Fusarium* nail infection: report of three cases. Br J Dermatol. 1997;136 (3): 424-7.
79. Krcmery V, Jr., Jesenska Z, Spanik S, Gyarfas J, Nogova J, Botek R, et al. Fungaemia due to *Fusarium* spp. in cancer patients. J Hosp Infect. 1997;36 (3): 223-8.
80. Kumar RR, Kumar BR, Shafiulla M, Lakshmaiah KC, Sridhar H. *Fusarium solani* infection in a patient with acute myelogenous leukemia-a case report. Indian J Pathol Microbiol. 1997;40 (4): 555-7.
81. Guinvarc'h A, Guilbert L, Marmorat-Khuong A, Lavarde V, Chevalier P, Amrein C, et al. Disseminated *Fusarium solani* infection with endocarditis in a lung transplant recipient. Mycoses. 1998; 41 (1-2): 59-61.
82. Sander A, Beyer U, Amberg R. Systemic *Fusarium oxysporum* infection in an immunocompetent patient with an adult respiratory distress syndrome (ARDS) and extracorporal membrane oxygenation (ECMO). Mycoses. 1998;41 (3-4): 109-11.
83. Camin AM, Michelet C, Langanay T, de Place C, Chevrier S, Gueho E, et al. Endocarditis due to *Fusarium dimerum* four years after coronary artery bypass grafting. Clin Infect Dis. 1999;28 (1): 150.
84. Girardi M, Glusac EJ, Imaeda S. Subcutaneous *Fusarium* foot abscess in a renal transplant patient. Cutis. 1999;63 (5): 267-70.
85. Goldblum D, Frueh BE, Zimmerli S, Bohnke M. Treatment of postkeratitis *Fusarium* endophthalmitis with amphotericin B lipid complex. Cornea. 2000;19 (6): 853-6.
86. Guarro J, Nucci M, Akiti T, Gene J, Barreiro MD, Goncalves RT. Fungemia due to *Fusarium sacchari* in an immunosuppressed patient. J Clin Microbiol. 2000;38 (1): 419-21.
87. Gupta AK, Baran R, Summerbell RC. *Fusarium* infections of the skin. Curr Opin Infect Dis. 2000;13 (2): 121-128.
88. Okada H, Hamatani S, Kondo M, Imai T, Itoh S, Isobe K, et al. Successful treatment of disseminated *Fusarium* infection in an infant with leukemia. Int J Hematol. 2000;72 (4): 494-8.
89. Peltroche-Llacsahuanga H, Manegold E, Kroll G, Haase G. Case report. Pathohistological findings in a clinical case of disseminated infection with *Fusarium oxysporum*. Mycoses. 2000;43 (9-10): 367-72.
90. Austen B, McCarthy H, Wilkins B, Smith A, Duncombe A. Fatal disseminated *Fusarium* infection in acute lymphoblastic leukaemia in complete remission. J Clin Pathol. 2001;54 (6): 488-90.
91. Pereiro M, Jr., Abalde MT, Zulaica A, Caeiro JL, Florez A, Peteiro C, et al. Chronic infection due to *Fusarium oxysporum* mimicking lupus vulgaris: case report and review of cutaneous involvement in fusariosis. Acta Derm Venereol. 2001;81 (1): 51-3.
92. Sampathkumar P, Paya CV. *Fusarium* infection after solid-organ transplantation. Clin Infect Dis. 2001;32 (8): 1237-40.
93. Letscher-Bru V, Campos F, Waller J, Randriamahazaka R, Candolfi E, Herbrecht R. Successful outcome of treatment of a disseminated infection due to *Fusarium dimerum* in a leukemia patient. J Clin Microbiol. 2002;40 (3): 1100-2.
94. Bader M, Jafri AK, Krueger T, Kumar V. *Fusarium* osteomyelitis of the foot in a patient with diabetes mellitus. Scand J Infect Dis. 2003;35 (11-12): 895-6.
95. Consigny S, Dhedin N, Datry A, Choquet S, Leblond V, Chosidow O. Successsful voriconazole treatment of disseminated *Fusarium* infection in an immunocompromised patient. Clin Infect Dis. 2003;37 (2): 311-3.
96. Durand-Joly I, Alfandari S, Benchikh Z, Rodrigue M, Espinel-Ingroff A, Catteau B, et al. Successful outcome of disseminated *Fusarium* infection with skin localization treated with voriconazole and amphotericin B-lipid complex in a patient with acute leukemia. J Clin Microbiol. 2003;41 (10): 4898- 900.
97. Guarro J, Rubio C, Gene J, Cano J, Gil J, Benito R, et al. Case of keratitis caused by an uncommon *Fusarium* species. J Clin Microbiol. 2003;41 (12): 5823-6.
98. Khoury H, Ball NJ. Disseminated fusariosis in a patient with acute leukaemia. Br J Haematol. 2003.120 (1): 1.
99. Mansoory D, Roozbahany NA, Mazinany H, Samimagam A. Chronic *Fusarium* infection in an adult patient with undiagnosed chronic granulomatous disease. Clin Infect Dis. 2003;37 (7): e107-8.
100. Rodriguez CA, Lujan-Zilbermann J, Woodard P, Andreansky M, Adderson EE. Successful treatment of disseminated fusariosis. Bone Marrow Transplant. 2003.31 (5): 411-2.
101. Vincent AL, Cabrero JE, Greene JN, Sandin RL. Successful voriconazole therapy of disseminated *Fusarium solani* in the brain of a neutropenic cancer patient. Cancer Control. 2003;10 (5): 414-9.
102. Albisetti M, Lauener RP, Gungor T, Schar G, Niggli FK, Nadal D. Disseminated *Fusarium oxysporum* infection in hemophagocytic lymphohistiocytosis. Infection. 2004;32 (6): 364-6.
103. Bigley VH, Duarte RF, Gosling RD, Kibbler CC, Seaton S, Potter M. *Fusarium dimerum* infection in a stem cell transplant recipient treated successfully with voriconazole. Bone Marrow Transplant. 2004;34 (9): 815-7.
104. Guzman-Cottrill JA, Zheng X, Chadwick EG. *Fusarium solani* endocarditis successfully treated with liposomal amphotericin B and voriconazole. Pediatr Infect Dis J. 2004;23 (11): 1059-61.
105. Herbrecht R, Kessler R, Kravanja C, Meyer MH, Waller J, Letscher-Bru V. Successful treatment of *Fusarium proliferatum* pneumonia with posaconazole in a lung transplant recipient. J Heart Lung Transplant. 2004; (12): 1451-4.
106. Kivivuori SM, Hovi L, Vettenranta K, Saarinen-Pihkala UM. Invasive fusariosis in two transplanted children. Eur J Pediatr. 2004;163 (11): 692-3.
107. Rothe A, Seibold M, Hoppe T, Seifert H, Engert A, Caspar C, et al. Combination therapy of disseminated *Fusarium oxysporum* infection with terbinafine and amphotericin B. Ann Hematol. 2004;83 (6): 394-7.
108. Anandi V, Vishwanathan P, Sasikala S, Rangarajan M, Subramaniyan CS, Chidambaram N. *Fusarium solani* breast abscess. Indian J Med Microbiol. 2005;23 (3): 198-9.
109. Cudillo L, Girmenia C, Santilli S, Picardi A, Dentamaro T, Tendas A, et al. Breakthrough fusariosis in a patient with acute lymphoblastic leukemia receiving voriconazole prophylaxis. Clin Infect Dis. 2005;40 (8): 1212-3.
110. Durand ML, Kim IK, D'Amico DJ, Loewenstein JI, Tobin EH, Kieval SJ, et al. Successful treatment of *Fusarium* endophthalmitis with voriconazole and *Aspergillus* endophthalmitis with voriconazole plus caspofungin. Am J Ophthalmol. 2005;140 (3): 552-4.
111. Hattori N, Shirai A, Sugiura Y, Li W, Yokoyama K, Misawa Y, et al. Onychomycosis caused by *Fusarium proliferatum*. Br J Dermatol. 2005;153 (3): 647-9.
112. Makowsky MJ, Warkentin DI, Savoie ML. Caspofungin and amphotericin B for disseminated *Fusarium verticillioides* in leukemia. Ann Pharmacother. 2005; 39 (7-8): 1365-6.
113. Rezai KA, Eliott D, Plous O, Vazquez JA, Abrams GW. Disseminated *Fusarium* infection presenting as bilateral endogenous endophthalmitis in a patient with acute myeloid leukemia. Arch Ophthalmol. 2005;123 (5): 702-3.
114. Dornbusch HJ, Buzina W, Summerbell RC, Lass-Florl C, Lackner H, Schwinger W, et al. *Fusarium verticillioides* abscess of the nasal septum in an immunosuppressed child: case report and identification of the morphologically atypical fungal strain. J Clin Microbiol. 2005;43 (4): 1998-2001.
115. Alfonso EC, Cantu-Dibildox J, Munir WM, Miller D, O'Brien TP, Karp CL, et al. Insurgence of *Fusarium* keratitis associated with contact lens wear. Arch Ophthalmol. 2006;124 (7): 941-7.
116. Cudillo L, Tendas A, Picardi A, Dentamaro T, Del Principe MI, Amadori S, et al. Successful treatment of disseminated fusariosis with high dose liposomal amphotericin-B in a patient with acute lymphoblastic leukemia. Ann Hematol. 2006;85 (2): 136-8.
117. Giaconi JA, Marangon FB, Miller D, Alfonso EC. Voriconazole and fungal keratitis: a report of two treatment failures. J Ocul Pharmacol Ther. 2006;22 (6): 437-9.
118. Sagnelli C, Fumagalli L, Prigitano A, Baccari P, Magnani P, Lazzarin A. Successful voriconazole therapy of disseminated *Fusarium verticillioides* infection in an immunocompromised patient receiving chemotherapy. J Antimicrob Chemother. 2006;57 (4): 796-8.
119. Chi CC, Wang SH. Disseminated cutaneous *Fusarium moniliforme* infections in a leukemic child. Int J Dermatol. 2007;46 (5): 487-9.
120. Nakamura Y, Xu X, Saito Y, Tateishi T, Takahashi T, Kawachi Y, et al. Deep cutaneous infection by *Fusarium solani* in a healthy child: successful treatment with local heat therapy. J Am Acad Dermatol. 2007;56 (5):873-7.
121. Ruiz N, Fernandez-Martos C, Romero I, Pla A, Maiquez J, Calatrava A, et al. Invasive fungal infection and nasal septum perforation with bevacizumab-based therapy in advanced colon cancer. J Clin Oncol. 2007;25 (22): 3376-7.
122. Tu EY, McCartney DL, Beatty RF, Springer KL, Levy J, Edward D. Successful treatment of resistant ocular fusariosis with posaconazole (SCH-56592). Am J Ophthalmol. 2007;143 (2): 222-227.
123. Anten S, Heddema ER, Visser O, Zweegman AS. Images in haematology. Cerebral fungal abscess in a patient with acute promyelocytic leukaemia. Br J Haematol. 2008 ;140(3):253.
124. Bordeaux JS, O'Brien M, Mahalingam M, Wiss K. Clinicopathologic challenge. Disseminated fusariosis. Int J Dermatol. 2008; 47(1):13-4.
125. Neuburger S, Massenkeil G, Seibold M, Lutz C, Tamm I, le Coutre P, et al. Successful salvage treatment of disseminated cutaneous fusariosis with liposomal amphotericin B and terbinafine after allogeneic stem cell transplantation. Transpl Infect Dis. 2008 ;10(4):290-3.
126. Ng AS, Lau WW, Yu DK, Wong CC, Chan CW. Clinical features and outcomes of *Fusarium* keratitis associated with contact lens wear. Eye Contact Lens. 2008;34(2):113-6.
127. de Magalhães Lima K, Machado Barbosa de Castro CM, Fonsêca Nogueira Cambuim II, Carvalhaes de Oliveira J, Delgado M, Sette de Melo Rego R. Non-dermatophytic moulds: onychomycosis in four patients infected with the human immunodeficiency virus. Rev Iberoam Micol. 2008;25(1):45-9.
128. Testerman GM, Steagald MK, Colquitt LA, Maki A. Disseminated *Fusarium* infection in a multiple trauma patient. South Med J. 2008;101(3):320-3.
129. Rekha A, Kindo AJ, Ravi A. *Fusarium solani* in the post-transplant patient: an unusual fungus. Int J Low Extrem Wounds. 2008;7(1):38-40.
130. Augsten R, Dawczynski J, Pfister W, Codina C, Königsdörffer E. *Fusarium* keratitis. Ophthalmologe. 2008;105(11):1043-5.
131. Kaufmann C, Frueh BE, Messerli J, Bernauer W, Thiel MA. Contact lens-associated *Fusarium* keratitis in Switzerland. Klin Monbl Augenheilkd. 2008; 225(5):418-21.
132. Seyfarth F, Ziemer M, Sayer HG, Burmester A, Erhard M, Welker M, et al. The use of ITS DNA sequence analysis and MALDI-TOF mass spectrometry in diagnosing an infection with *Fusarium proliferatum*. Exp Dermatol. 2008;17(11):965-71.
133. Miller WL, Giannoni AG, Perrigin J. A case of fungal keratitis: a clinical and *in vivo* confocal microscopy assessment. Cont Lens Anterior Eye. 2008 ;31(4):201-6.
134. Macêdo DP, Neves RP, Fontan J, Souza-Motta CM, Lima D. A case of invasive rhinosinusitis by *Fusarium verticillioides* (Saccardo) Nirenberg in an apparently immunocompetent patient. Med Mycol. 2008;46(5):499-503.
135. Tran TH, Terrada C. Perivascular granular deposits following ocular toxoplasmosis in a systemic B cell lymphoma-immunodepressed patient. J Fr Ophtalmol. 2008;31(5):557-9.
136. Sauer A, Abry F, Lhermitte B, Candolfi E, Speeg-Schatz C, Bourcier T. Purulent corneal melting secondary to multidrug-resistant *Fusarium oxysporum* aggravated by topical corticosteroid therapy. J Fr Ophtalmol. 2008;31(5):534.e15.
137. Hermansen NE, Ralfkiaer EM, Kjeldsen L. Disseminated fusariosis in a patient with acute lymphoblastic leukaemia. Ugeskr Laeger. 2008 8;170(37):2892.
138. Saltzmann RM, Yep JM, Blomquist PH. *Fusarium* keratitis associated with ReNu with MoistureLoc sample kits. Eye Contact Lens. 2008;34(6):337-9.
139. Mallo-García S, Coto-Segura P, Santos-Juanes-Jiménez J. Proximal white subungual onychomycosis due to *Fusarium* species. Actas Dermosifiliogr. 2008; 99(9):742-3.
140. Brasch J, Köppl G. Persisting onychomycosis caused by *Fusarium solani* in an immunocompetent patient. Mycoses. 2009;52(3):285-6.
141. Tezcan G, Ozhak-Baysan B, Alastruey-Izquierdo A, Ogunc D, Ongut G, Yildiran ST, et al. Disseminated fusariosis caused by *Fusarium verticillioides* in an acute lymphoblastic leukemia patient after allogeneic hematopoietic stem cell transplantation. J Clin Microbiol. 2009 ;47(1):278-81.
142. Lin HC, Hsiao CH, Ma DH, Yeh LK, Tan HY, Lin MY, Huang SC. Medical treatment for combined *Fusarium* and *Acanthamoeba* keratitis. Acta Ophthalmol. 2009 87(2):199-203.
143. Busemann C, Krüger W, Schwesinger G, Kallinich B, Schröder G, Abel P, et al. Myocardial and aortal involvement in a case of disseminated infection with *Fusarium solani* after allogeneic stem cell transplantation: report of a case. Mycoses. 2009;52(4):372-6.
144. Avisar I, Weinberger D, Kremer I. *Fusarium* keratitis and endophthalmitis treated by intravenous ambisome. Harefuah. 2009;148(1):28-9.
145. Peponis V, Rosenberg P, Chalkiadakis SE, Insler M, Amariotakis A. Fungal scleral keratitis and endophthalmitis following pterygium excision. Eur J Ophthalmol. 2009;19(3):478-80.
146. Tascini C, Urbani L, Doria R, Catalano G, Leonildi A, Filipponi F, Menichetti F. Breakthrough *Fusarium* spp fungemia during caspofungin therapy in an ABO-incompatible orthotopic liver transplant patient. J Chemother. 2009; 21(2):236-8.
147. Cooke NS, Feighery C, Armstrong DK, Walsh M, Dempsey S. Cutaneous *Fusarium solani* infection in childhood acute lymphoblastic leukaemia. Clin Exp Dermatol. 2009;34(5):e117-9.
148. Patterson TF, Mackool BT, Gilman MD, Piris A. Case records of the Massachusetts General Hospital. Case 22-2009. A 59-year-old man with skin and pulmonary lesions after chemotherapy for leukemia. N Engl J Med. 2009;361(3):287-96.
149. Cuellar-Rodriguez J, Bravo LT, Oethinger M, Fraser T, Mossad SB. Disseminated fusariosis in a recipient of a bone-marrow transplant. Lancet Infect Dis. 2009;9(8):520.
150. Aggermann T, Haas P, Krepler K, Binder S, Hochwarter A*. Fusarium* endophthalmitis following refractive lens exchange for correction of high myopia. J Cataract Refract Surg. 2009;35(8):1468-70.
151. Spriet I, Delaere L, Lagrou K, Peetermans WE, Maertens J, Willems L. Intraocular penetration of voriconazole and caspofungin in a patient with fungal endophthalmitis. J Antimicrob Chemother. 2009;64(4):877-8.
152. Menon BS, Juraida E, Manaf Z, Mohamed M, Ibrahim H. Disseminated *Fusarium*. Int J Infect Dis. 2009 ;13(5):e333-4.
153. Yang YS. Results of extensive surgical treatment of seven consecutive cases of postoperative fungal endophthalmitis. Korean J Ophthalmol. 2009;23(3):159-63.
154. Müller C, Schumacher U, Gregor M, Lamprecht G. How immunocompromised are short bowel patients receiving home parenteral nutrition? Apropos a case of disseminated *Fusarium oxysporum* sepsis. JPEN J Parenter Enteral Nutr. 2009;33(6):717-20.
155. Kleinschmidt-Demasters BK. Disseminated *Fusarium* infection with brain abscesses in a lung transplant recipient. Clin Neuropathol. 2009;28(6):417-21.
156. Touvron G, Denis D, Doat M, Girard A, Brandely ML, Chast F, et al. Successful treatment of resistant *Fusarium solani* keratitis with liposomal amphotericin B. J Fr Ophtalmol. 2009;32(10):721-6.
157. Zhang CZ, Fung MA, Eisen DB. Disseminated fusariosis presenting as panniculitis-like lesions on the legs of a neutropenic girl with acute lymphoblastic leukemia. Dermatol Online J. 2009;15(10):5.
158. Cakir M, Imamoğlu S, Cekiç O, Bozkurt E, Alagöz N, Oksüz L, et al. An outbreak of early-onset endophthalmitis caused by *Fusarium* species following cataract surgery. Curr Eye Res. 2009;34(11):988-95.
159. Wu CY, Chen GS, Lan CC. Onychomycosis caused by *Fusarium solani* in a woman with diabetes. Clin Exp Dermatol. 2009;34(8):e772-4.
160. Cesaro S, Marinello S, Alessia B, Alaggio R, Rossi L, Toffolutti T, et al, Successful treatment of disseminated fusariosis in a child with acute myelogenous leukaemia with medical and surgical approach. Mycoses. 2010;53(2):181-5.
161. Jossi M, Ambrosioni J, Macedo-Vinas M, Garbino J. Invasive fusariosis with prolonged fungemia in a patient with acute lymphoblastic leukemia: case report and review of the literature. Int J Infect Dis. 2010;14(4):e354-6.
162. Palmore TN, Shea YR, Childs RW, Sherry RM, Walsh TJ. *Fusarium proliferatum* soft tissue infection at the site of a puncture by a plant: recovery, isolation, and direct molecular identification. J Clin Microbiol. 2010;48(1):338-42.
163. Lee B, Grossniklaus HE, Edelhauser HF. Concurrent *acanthamoeba* and *Fusarium* keratitis with silicone hydrogel contact lens use. Cornea. 2010;29(2):210-3.
164. Kantarcioglu AS, Summerbell RC, Sutton DA, Yücell A, Sarikaya E, Kaner et al. A dark strain in the *Fusarium solani* species complex isolated from primary subcutaneous sporotrichioid lesions associated with traumatic inoculation via a rose bush thorn. Med Mycol. 2010;48(1):103-9.
165. Cesaro S, Marinello S, Alessia B, Alaggio R, Rossi L, Toffolutti T, et al. Successful treatment of disseminated fusariosis in a child with acute myelogenous leukaemia with medical and surgical approach. Mycoses. 2010;53(2):181-5.
166. Jossi M, Ambrosioni J, Macedo-Vinas M, Garbino J. Invasive fusariosis with prolonged fungemia in a patient with acute lymphoblastic leukemia: case report and review of the literature. Int J Infect Dis. 2010;14(4):e354-6.
167. Baudraz-Rosselet F, Ruffieux C, Lurati M, Bontems O, Monod M. Onychomycosis insensitive to systemic terbinafine and azole treatments reveals non-dermatophyte moulds as infectious agents. Dermatology. 2010;220(2):164-8.
168. Romano C, Caposciutti P, Ghilardi A, Miracco C, Fimiani M. A case of primary localized cutaneous infection due to *Fusarium oxysporum*. Mycopathologia. 2010;170(1):39-46.
169. Halpern M, Balbi E, Carius L, Roma J, Gonzalez AC, Agoglia L, et al. Cellulitis and nodular skin lesions due to *Fusarium* spp in liver transplant: case report. Transplant Proc. 2010;42(2):599-600.
170. Gaur S, Rajgopal A, Ashbee R. A successfully treated case of peritonitis due to *Fusarium dimerum*. J Infect. 2010;61(1):86-8.
171. Huang WW, Gray C, Bowers R, Walk N, Lind A, Hornstra I. Painful necrotic nodules in an immunocompromised patient. Arch Dermatol. 2010;146(4):439-44.
172. Chen QX, Li CX, Huang WM, Shi JQ, Li SF. Cutaneous hyalohyphomycosis caused by *Fusarium subglutinans*. Eur J Dermatol. 2010;20(4):526-7.
173. Bourgeois GP, Cafardi JA, Sellheyer K, Andea AA. Disseminated *Fusarium* infection originating from paronychia in a neutropenic patient: a case report and review of the literature. Cutis. 2010;85(4):191-4.
174. Zaigraykina N, Tomkins O, Garzozi HJ, Potasman I. *Fusarium* keratitis acquired during travel to Namibia. J Travel Med. 2010;17(3):209-11.
175. Richetta AG, Lichtener M, Mattozzi C, Miccoli A, Giancristoforo S, D'Epiro S, et al. Fusariosis and skin T cell lymphoma: concomitant more than a differential diagnosis. Clin Ter. 2010;161(3):265-7.
176. Garcia-Delpech S, Díaz-Llopis M, Udaondo P, Salom D. *Fusarium* keratitis 3 weeks after healed corneal cross-linking. J Refract Surg. 2010;26(12):994-5
177. Takezawa Y, Shiraishi A, Noda E, Hara Y, Yamaguchi M, Uno T, et al. Effectiveness of *in vivo* confocal microscopy in detecting filamentous fungi during clinical course of fungal keratitis. Cornea. 2010;29(12):1346-52.
178. Rieger KE, Ridky TW, Sundram UN. Skin nodules in a patient with acute myeloid leukemia and neurological deterioration-quiz case. Disseminated fusariosis. Arch Dermatol. 2010;146(9):1037-42.
179. Zribi J, Boudaya S, Sallemi A, Masmoudi A, Chaabène H, Makni F, et al. Atypical cutaneous *Fusarium* infection in an immunocompetent patient. Ann Dermatol Venereol. 2010 ;137(10):630-4.
180. Mellouli F, Ksouri H, Barbouche R, Maamer M, Hamed LB, Hmida Set al. Successful treatment of *Fusarium solani* ecthyma gangrenosum in a patient affected by leukocyte adhesion deficiency type 1 with granulocytes transfusions. BMC Dermatol. 2010;10:10.
181. Proença-Pina J, Ssi Yan Kai I, Bourcier T, Fabre M, Offret H, Labetoulle M. *Fusarium* keratitis and endophthalmitis associated with lens contact wear. Int Ophthalmol. 2010 ;30(1):103-7.
182. Mansur AT, Artunkal S, Ener B. *Fusarium oxysporum* infection of stasis ulcer: eradication with measures aimed to improve stasis.Mycoses. 2011;54(4):e2057.
183. Yang YS, Ahn JJ, Shin MK, Lee MH. *Fusarium solani* onychomycosis of the thumbnail coinfected with Pseudomonas aeruginosa: report of two cases. Mycoses. 2011;54(2):168-71.
184. Nunes Mdo C, Barbosa FB, Gomes GH, Bráulio R, Nicoliello MF, Ferrari TC. Fatal right-sided endocarditis caused by *Fusarium* in an immunocompromised patient: a case report. Mycoses. 2011 Sep;54(5):460-2.
185. Chander J, Singla N, Gulati N, Sood S. *Fusarium sacchari*: a cause of exogenous fungal endophthalmitis: first case report and review of literature. Mycopathologia. 2011;171(6):431-4.
186. Carrasco MA, Genesoni G. Treatment of severe fungal keratitis with subconjunctival amphotericin B. Cornea. 2011;30(5):608-11.
187. Carneiro HA, Coleman JJ, Restrepo A, Mylonakis E. *Fusarium* infection in lung transplant patients: report of 6 cases and review of the literature. Medicine (Baltimore). 2011 Jan;90(1):69-80.
188. Bose P, Parekh HD, Holter JL, Greenfield RA. Disseminated fusariosis occurring in two patients despite posaconazole prophylaxis. J Clin Microbiol. 2011 ;49(4):1674-5.
189. Kapp M, Schargus M, Deuchert T, Springer J, Wendel F, Loeffler J, et al. Endophthalmitis as primary clinical manifestation of fatal fusariosis in an allogeneic stem cell recipient. Transpl Infect Dis. 2011;13(4):374-9.
190. Gurusidappa SB, Mamatha HS. Fusarial skin lesion in immunocompromised. Indian J Cancer. 2011;48(1):116-7.
191. Salas-Coronas J, Cabezas-Fernández T, Martínez-Lage MJ, Villarejo-Ordóñez A. Mycetoma caused by *Fusarium solani*. Rev Clin Esp. 2011;211(3):e16-8.
192. Labbé A, Gabison E, Cochereau I, Baudouin C. Diagnosis of fungal keratitis by *in vivo* confocal microscopy: a case report. Eye (Lond). 2011;25(7):956-8.
193. Siatiri H, Daneshgar F, Siatiri N, Khodabande A. The effects of intrastromal voriconazole injection and topical voriconazole in the treatment of recalcitrant *Fusarium* keratitis. Cornea. 2011;30(8):872-5.
194. Edupuganti S, Rouphael N, Mehta A, Eaton M, Heller JG, Bressler A, et al. *Fusarium falciforme* vertebral abscess and osteomyelitis: case report and molecular classification. J Clin Microbiol. 2011;49(6):2350-3.
195. Liu JY, Chen WT, Ko BS, Yao M, Hsueh PR, Hsiao CH, et al. Combination antifungal therapy for disseminated fusariosis in immunocompromised patients: a case report and literature review. Med Mycol. 2011;49(8):872-8.
196. Thomas PA, Jesudasan CA, Geraldine P, Kaliamurthy J. Adventitious sporulation in *Fusarium* keratitis. Graefes Arch Clin Exp Ophthalmol. 2011; 249 (9):1429-31.
197. Terada M, Fujita J, Watanabe S, Kawasaki M, Tanabe H, Anzawa K, et al. Olecranon bursa with *Fusarium solani* infection in an otherwise healthy patient. Mycoses. 2011;54(6):e853-5.
198. Banerji JS, Singh J C. Cutaneous *Fusarium* infection in a renal transplant recipient: a case report. J Med Case Rep. 2011;5:205.
199. Güngel H, Eren MH, Pınarcı EY, Altan C, Baylançiçek DO, Kara N, et al. An outbreak of *Fusarium solani* endophthalmitis after cataract surgery in an eye training and research hospital in Istanbul. Mycoses. 2011;54(6):e767-74.
200. Dai W, Dharamsi JW, Soliman S, Ricotti C, Gander R, Bergstresser P, et al. Cutaneous fusariosis developing in a post-irradiation site. Dermatol Online J. 2011;17(5):5.
201. Kano R, Maruyama H, Kubota M, Hasegawa A, Kamata H. Chronic ulcerative dermatitis caused by *Fusarium sporotrichioides*. Med Mycol. 2011;49(3):303-5.
202. King BA, Seropian S, Fox LP. Disseminated *Fusarium* infection with muscle involvement. J Am Acad Dermatol. 2011;65(1):235-7.
203. Gutiérrez Paredes EM, Gámez Pérez L, González Rodríguez AJ, Ramón Quiles D, Monteagudo Castro C, Jordá Cuevas E. Disseminated fusariosis in immunocompromised patients. Eur J Dermatol. 2011;21(5):753-5
204. Gilaberte Y, Aspiroz C, Martes MP, Alcalde V, Espinel-Ingroff A, Rezusta A. Treatment of refractory fingernail onychomycosis caused by nondermatophyte molds with methylaminolevulinate photodynamic therapy. J Am Acad Dermatol. 2011 ;65(3):669-71.
205. Cocchi S, Codeluppi M, Venturelli C, Bedini A, Grottola A, Gennari W, et al. *Fusarium verticillioides* fungemia in a liver transplantation patient: successful treatment with voriconazole. Diagn Microbiol Infect Dis. 2011;71(4):438-41.
206. Labois A, Gray C, Lepretre S. Successful treatment of disseminated fusariosis with voriconazole in an acute lymphoblastic leukaemia patient. Mycoses. 2011;4:8-11.
207. Lurati M, Baudraz-Rosselet F, Vernez M, Spring P, Bontems O, Fratti M, et al. Efficacious treatment of non-dermatophyte mould onychomycosis with topical amphotericin B. Dermatology. 2011;223(4):289-92.
208. Mikulska M, Furfaro E, Del Bono V, Gualandi F, Raiola AM, Molinari MP, et al. Galactomannan testing might be useful for early diagnosis of fusariosis. Diagn Microbiol Infect Dis. 2012;72(4):367-9.
209. Mochizuki K, Shiraki I, Murase H, Ohkusu K, Nishimura K. Identification and sensitivity of two rare fungal species isolated from two patients with *Fusarium* keratomycosis. J Infect Chemother. 2012;18(6):939-44.
210. Taylan Sekeroglu H, Erdem E, Yagmur M, Gumral R, Ersoz R, Ilkit M, Harbiyeli II. Successful medical management of recalcitrant *Fusarium solani* keratitis: molecular identification and susceptibility patterns. Mycopathologia. 2012 ;174(3):233-7.
211. Edelstein SL, Akduman L, Durham BH, Fothergill AW, Hsu HY. Resistant *Fusarium* keratitis progressing to endophthalmitis. Eye Contact Lens. 2012;38(5):331-5.
212. Brasch J, Shimanovich I. Persistent fingernail onychomycosis caused by *Fusarium proliferatum* in a healthy woman. Mycoses. 2012;55(1):86-9.
213. Park KY, Lee JW, Kim IS, Suh MK, Choi JS, Moon NJ, Seo SJ. Longitudinal melanonychia caused by *Fusarium oxysporum* in immunocompetent patient. Mycoses. 2012;55(2):e40-1.
214. Brasch J, Beck-Jendroschek V, Wohlfeil E. Recalcitrant purulent paronychia and onychomycosis caused by *Fusarium oxysporum*. J Dtsch Dermatol Ges. 2012;10(7):519-20.
215. Singhal KV, Saoji V, Saoji SV. *Fusarium* skin infection: a case report. Dermatol Online J. 2012;18(4):6.
216. Kawashima N, Yoshida N, Matsushita N, Ito M, Matsumoto K, Kato K. Intra-articular injection of voriconazole for *Fusarium solani* arthritis after bone marrow transplantation. J Infect. 2012;65(4):366-7.
217. Behrens-Baumann W, Seibold M, Hofmüller W, Walter S, Haeberle H, Wecke T, Tammer I, Tintelnot K. Benefit of polyhexamethylene biguanide in *Fusarium* keratitis. Ophthalmic Res. 2012;48(4):171-6.
218. Labiris G, Troeber L, Gatzioufas Z, Stavridis E, Seitz B. Bilateral *Fusarium oxysporum* keratitis after laser in situ keratomileusis. J Cataract Refract Surg. 2012;38(11):2040-4.
219. Zegarra-Linares R, Wang ZJ, Lephart P, Poulik J, Rongkavilit C. A knee ulcer in a teenager with acute myeloid leukemia. Pediatr Infect Dis J. 2012; 31(10):1100, 1104-5.
220. Khan S, Pillai GS, Vivek V, Dinesh K, Karim PM. Post-operative endophthalmitis due to *Fusarium dimerum*. Southeast Asian J Trop Med Public Health. 2012;43(6):1484-8.
221. Morel LN, Cid PM, De Celada RM, Rodríguez MF, Beato M, Arias ÁG, et al. Disseminated fusariosis in a pediatric population. Pediatr Dermatol. 2013;30(6):e255-6.
222. Pereira GH, de Angelis DA, Brasil RA, dos Anjos Martins M, de Matos Castro e Silva D, Szeszs MW, et al. Disseminated amphotericin-resistant fusariosis in acute leukemia patients: report of two cases. Mycopathologia. 2013;175(1-2):107-14.
223. Fanci R, Pini G, Bartolesi AM, Pecile P. Refractory disseminated fusariosis by *Fusarium verticillioides* in a patient with acute myeloid leukaemia relapsed after allogeneic hematopoietic stem cell transplantation: a case report and literature review. Rev Iberoam Micol. 2013;30(1):51-3.
224. Pellegrino F, Carrasco MA. Argon laser phototherapy in the treatment of refractory fungal keratitis. Cornea. 2013;32(1):95-7.
225. Collado C, Medina L, Zorraquino A, Baeza T, Ferrer C, Plazas J, et al. Cutaneous fusariosis by a species of the *Fusarium dimerum* species complex in a patient with acute myeloblastic leukemia. Rev Iberoam Micol. 2013;30(2):119-21.
226. Yan X, Yu C, Shi Z, Wang S, Zhang F. Nasal cutaneous infection in a healthy boy caused by *Fusarium moniliforme*. Pediatr Dermatol. 2013;30(4):e43-5.
227. Rajmane VS, Rajmane ST, Patil VC, Patil AB, Mohite ST. Maxillary rhinosinusitis due to *Fusarium* species leading to cavernous sinus thrombosis. J Mycol Med. 2013;23(1):53-6.
228. Calcaterra D, Karam K, Suzuki Y. Computed tomography findings in a patient with fungal aortitis: acute aortic syndrome secondary to fusariosis. Interact Cardiovasc Thorac Surg. 2013;17(1):171-2.
229. Inano S, Kimura M, Iida J, Arima N. Combination therapy of voriconazole and terbinafine for disseminated fusariosis: case report and literature review. J Infect Chemother. 2013;19(6):1173-80.
230. Kang Y, Li L, Zhu J, Zhao Y, Zhang Q. Identification of *Fusarium* from a patient with fungemia after multiple organ injury. Mycopathologia. 2013;176(1-2):151-5
231. Bouanani N, Lamchahab M, Quachouh M, Soussi M, Quessar A, Benchekroun S. Disseminated fusariosis during autologous stem cells transplant. J Mycol Med. 2013;23(2):119-22.
232. Silva GM, Silveira AR, Betânia CA, Macêdo DP, Neves RP. Disseminated fusariosis secondary to neuroblastoma with fatal outcome. Mycopathologia. 2013; 176(3-4):233-6. doi: 10.1007/s11046-013-9674-8.
233. Dutta P, Premkumar A, Chakrabarti A, Shah VN, Behera A, De D, et al. *Fusarium falciforme* infection of foot in a patient with type 2 diabetes mellitus: a case report and review of the literature. Mycopathologia. 2013;176(3-4):225-32.
234. Colombo A, Maccari G, Congiu T, Basso P, Baj A, Toniolo A. Colonization of a Central Venous Catheter by the Hyaline Fungus *Fusarium solani* Species Complex: A Case Report and SEM Imaging. Case Rep Med. 2013:618358.
235. Perini GF, Camargo LF, Lottenberg CL, Hamerschlak N. Disseminated fusariosis with endophthalmitis in a patient with hematologic malignancy. Einstein (Sao Paulo). 2013;11(4):545-6.
236. Sidhu S, Chander J, Singh K. Perinephric abscess caused by *Fusarium chlamydosporum* in an immunocompetent child: case report and identification of the morphologically atypical fungal strain. Indian J Pathol Microbiol. 2013; 56(3):312-4.
237. Cavallini GM, Ducange P, Volante V, Benatti C. Successful treatment of *Fusarium* keratitis after photo refractive keratectomy. Indian J Ophthalmol. 2013; 61(11):669-71.
238. Jiang K, Brownstein S, Baig K, Lam K, Toye B. Clinicopathologic case reports of Alternaria and *Fusarium* keratitis in Canada. Can J Ophthalmol. 2013 ;48(6):e151-4.
239. Liu YS, Wang NC, Ye RH, Kao WY. Disseminated *Fusarium* infection in a patient with acute lymphoblastic leukemia: A case report and review of the literature. Oncol Lett. 2014;7(2):334-336.
240. Alnawaiseh M, Böhm MR, Idelevich EA, Becker K, Grewe S, Grenzebach UH, et al. Successful treatment of *Fusarium*-associated keratitis with multiresistant pathogen and multimorbid patient. Ophthalmologe. 2014;111(3):259-61.
241. Wu CH, Lu PL, Hsiao HH, Liu TC, Lin SF, Chang CS, et al. Breakthrough *Fusarium solani* infection in a patient with acute myeloid leukemia receiving posaconazole prophylaxis. Ann Hematol. 2014;93(6):1079-81.
242. Le Clech L, Hutin P, Le Gal S, Guillerm G. Skin nodules in a patient with acute lymphoblastic leukaemia. BMJ Case Rep. 9;2014.
243. Ikeda I, Ohno T, Ohno H, Miyazaki Y, Nishimoto K, Fukushima S, et al. Case of *Fusarium* paronychia successfully treated with occlusive dressing of antifungal cream. J Dermatol. 2014;41(4):340-2.
244. Kebabcı N, van Diepeningen AD, Ener B, Ersal T, Meijer M, Al-Hatmi AM, Ozkocaman V, et al. Fatal breakthrough infection with *Fusarium andiyazi*: new multi-resistant aetiological agent cross-reacting with Aspergillus galactomannan enzyme immunoassay. Mycoses. 2014;57(4):249-55.
245. Jørgensen JS, Prause JU, Kiilgaard JF. Bilateral endogenous *Fusarium solani* endophthalmitis in a liver-transplanted patient: a case report. J Med Case Rep. 2014 24;8:101.
246. Cheng P, Meng F, Zhang D. Fatal *Fusarium solani* infection after stem cell transplant for aplastic anemia. Exp Clin Transplant. 2014;12(4):384-7.
247. Terasaki JM, Shah SK, Schnadig VJ, Valentine VG. Airway complication contributing to disseminated fusariosis after lung transplantation. Transpl Infect Dis. 2014;16(4):621-4.
248. Keskar VS, Wanjare S, Jamale TE, Mahajan D, Jawale SY, Fernandes G, et al. Subcutaneous hyalohyphomycosis caused by *Fusarium* in a kidney transplant recipient. Ren Fail. 2014 Aug;36(7):1129-32.
249. Nakai K, Yoneda K, Imataki O, Kida J, Uemura M, Moriue T, et al. Transepidermal growth in disseminated *Fusarium* infection. J Dermatol. 2014; 41(8):770-1.
250. Shah PJ, Bergman S, Vegi S, Sundareshan V. *Fusarium* peritonitis successfully managed with posaconazole and catheter removal. Perit Dial Int. 2014; 34(5):566-8.
251. de Souza M, Matsuzawa T, Lyra L, Busso-Lopes AF, Gonoi T, Schreiber AZ, et al. *Fusarium napiforme* systemic infection: case report with molecular characterization and antifungal susceptibility tests. Springerplus. 2014;3:492.
252. Atty C, Alagiozian-Angelova VM, Kowal-Vern A. Black plaques and white nodules in a burn patient. *Fusarium* and Mucormycosis. JAMA Dermatol. 2014 Dec;150(12):1355-6.
253. Peterson A, Pham MH, Lee B, Commins D, Cadden J, Giannotta SL, et al. Intracranial *Fusarium* fungal abscess in an immunocompetent patient: case report and review of the literature. J Neurol Surg Rep. 2014;75(2):e241-5.
254. Al-Hatmi AM, Bonifaz A, de Hoog GS, Vazquez-Maya L, Garcia-Carmona K, Meis JF, van Diepeningen AD. Keratitis by *Fusarium temperatum*, a novel opportunist. BMC Infect Dis. 2014;14:588.
255. Antequera P, Garcia-Conca V, Martín-González C, Ortiz-de-la-Tabla V. Multidrug resistant *Fusarium* keratitis. Arch Soc Esp Oftalmol. 2015;90(8):382-4.
256. Garcia RR, Min Z, Narasimhan S, Bhanot N. *Fusarium* brain abscess: case report and literature review. Mycoses. 2015;58(1):22-6.
257. Agarwal T, Bandivadekar P, Satpathy G, Sharma N, Titiyal JS. Detection of fungal hyphae using smartphone and pocket magnifier: going cellular. Cornea. 2015; 34(3):355-7.
258. del Alcazar E, Jaka A, Camino N, Gancho G, Tuneu A. Fever and skin lesions in an immunocompromised patient. Clin Exp Dermatol. 2015;40(2):219-21.
259. Avelino-Silva VI, Ramos JF, Leal FE, Testagrossa L, Novis YS. Disseminated *Fusarium* infection in autologous stem cell transplant recipient. Braz J Infect Dis. 2015;19 (1):90-3.
260. Mena R, Carrasco E, Godoy-Martínez P, Stchigel AM, Cano-Lira JF, Zaror L. A case of mycotic keratitis due to *Fusarium solani* in Valdivia, Chile. Rev Iberoam Micol. 2015;32(2):106-10.
261. Dickson SD, Tankersley MS. Fatal Hypersensitivity Pneumonitis from Exposure to *Fusarium vasinfectum* in a Home Environment: A Case Report. Int Arch Allergy Immunol. 2015;166(2):150-3.
262. Al-Hatmi AM, Bonifaz A, Calderón L, Curfs-Breuker I, Meis JF, van Diepeningen AD, de Hoog GS. Proximal subungual onychomycosis caused by *Fusarium falciforme* successfully cured with posaconazole. Br J Dermatol. 2015; 173(1):253-5.
263. Kandeel A, Abu-Elmagd K, Spinner M, Khanna A, Hashimoto K, Fujiki M, et al. Atypical Clinical Presentation of a Newer Generation Anti-Fungal Drug-Resistant *Fusarium* Infection After a Modified Multi-Visceral Transplant. Ann Transplant. 2015;20:512-8.
264. Akers KS, Rowan MP, Niece KL, Graybill JC, Mende K, Chung KK, et al. Antifungal wound penetration of amphotericin and voriconazole in combat-related injuries: case report. BMC infectious diseases. 2015;15(1):184.
265. Ricna D, Lengerova M, Palackova M, Hadrabova M, Kocmanova I, Weinbergerova B, et al. Disseminated fusariosis by *Fusarium proliferatum* in a patient with aplastic anaemia receiving primary posaconazole prophylaxis - case report and review of the literature. Mycoses. 2016;59(1):48-55.
